# Proteomics, phylogenetics, and coexpression analyses indicate novel interactions in the plastid CLP chaperone-protease system

**DOI:** 10.1016/j.jbc.2022.101609

**Published:** 2022-01-20

**Authors:** Jui-Yun Rei Liao, Giulia Friso, Evan S. Forsythe, Elena J.S. Michel, Alissa M. Williams, Sasha S. Boguraev, Lalit Ponnala, Daniel B. Sloan, Klaas J. van Wijk

**Affiliations:** 1 Section of Plant Biology, School of Integrative Plant Sciences (SIPS), Cornell University, Ithaca, New York, USA; 2 Graduate Program in Cell and Molecular Biology, Department of Biology, Colorado State University, Fort Collins, Colorado, USA; 3 Viqstra, Inc, Staten Island, New York, USA

**Keywords:** CLP serine protease, AAA+ chaperone, domain of unknown function (DUF) proteins, adaptors, proteolysis, proteostasis, substrate trapping, chloroplast, *Arabidopsis thaliana*, ARM, armadillo repeat, DUF, domains of unknown function, ERC, Evolutionary Rate Covariation, GBP, glutamyl-tRNA reductase binding protein, GluTR, glutamyl-tRNA reductase, LS, logit score, MS/MS, tandem mass spectrometry, PPDB, Plant Proteome Database, SPC, spectral count, SPP, stromal processing peptidase

## Abstract

The chloroplast chaperone CLPC1 unfolds and delivers substrates to the stromal CLPPRT protease complex for degradation. We previously used an *in vivo* trapping approach to identify interactors with CLPC1 in *Arabidopsis thaliana* by expressing a STREPII-tagged copy of CLPC1 mutated in its Walker B domains (CLPC1-TRAP) followed by affinity purification and mass spectrometry. To create a larger pool of candidate substrates, adaptors, or regulators, we carried out a far more sensitive and comprehensive *in vivo* protein trapping analysis. We identified 59 highly enriched CLPC1 protein interactors, in particular proteins belonging to families of unknown functions (DUF760, DUF179, DUF3143, UVR-DUF151, HugZ/DUF2470), as well as the UVR domain proteins EXE1 and EXE2 implicated in singlet oxygen damage and signaling. Phylogenetic and functional domain analyses identified other members of these families that appear to localize (nearly) exclusively to plastids. In addition, several of these DUF proteins are of very low abundance as determined through the Arabidopsis PeptideAtlas http://www.peptideatlas.org/builds/arabidopsis/ showing that enrichment in the CLPC1-TRAP was extremely selective. Evolutionary rate covariation indicated that the HugZ/DUF2470 family coevolved with the plastid CLP machinery suggesting functional and/or physical interactions. Finally, mRNA-based coexpression networks showed that all 12 CLP protease subunits tightly coexpressed as a single cluster with deep connections to DUF760-3. Coexpression modules for other trapped proteins suggested specific functions in biological processes, *e.g.*, UVR2 and UVR3 were associated with extraplastidic degradation, whereas DUF760-6 is likely involved in senescence. This study provides a strong foundation for discovery of substrate selection by the chloroplast CLP protease system.

Plastids undergo developmental transitions from nonphotosynthetic plastids in roots to photosynthetic chloroplasts in green tissues and are able to adapt to (a)biotic conditions (1). Each plastid type must contain a specific proteome through the coordinated actions of the proteostasis network, involving transcription, translation, protein folding, and degradation machineries. The remodeling and stability of these proteomes during plastid differentiation and adaptation occurs through selective protein synthesis and proteolysis. Understanding the proteolytic hierarchies and degrons is therefore essential to understand plastid differentiation, adaptation, and function (2, 3, 4). The most abundant and complex protease system in the chloroplast is the soluble CLP system located in the stroma. Forward and/or reverse genetics in *Arabidopsis*, maize, rice, and tobacco demonstrated the essential nature of the plastid CLP system. Complete loss of the CLPC chaperone or CLPPR protease capacity results in embryo lethality, whereas partial loss results in delayed growth and development and virescent leaves (5, 6, 7).

The plastid CLP system in *Arabidopsis* consists of a hetero-oligomeric protease core comprising one or more copies of five proteolytically active subunits (CLPP1 and CLPP3-6), four proteolytically inactive proteins (CLPR1-4), as well as two plant-specific accessory proteins (CLPT1,2), three AAA+ chaperones (CLPC1, CLPC2, CLPD), and two adaptors CLPS1 and CLPF. Plastids do not contain any CLPX homologs, which instead are present in mitochondria along with CLPP2 (5). A recent study showed that there is a tight correlation between amino acid substitution rates in the plastid-encoded CLPP1 and the nuclear-encoded CLP subunits across a broad sampling of angiosperms, suggesting continuing selection on interactions within this complex (8).

CLP-dependent proteolysis is an ATP-dependent multistep regulated process that involves the CLP chaperones assembled into hexamers and the CLP protease core. The CLPC1,2 and CLPD chaperones have two ATPase domains and an IGF motif that is essential for binding to the CLP protease core complex (7). The CLPC chaperones accumulate as dimers when not engaged in the degradation cycle, and formation of the chaperone hexamer requires priming of the chaperone by adaptors and/or ATP leading to the formation of the active hexamer in the ATP-bound state (5, 9, 10). Substrates are recognized directly by the CLP chaperone(s) and/or by active recruitment by so-called adaptor proteins or recognins, or even other chaperones. Upon interaction of the substrate with the CLP chaperone, the ATP-dependent substrate unfolding process starts and the CLP protease core complex is recruited to the substrate–chaperone assembly. ATP binding and hydrolysis is required for substrate unfolding. In contrast, the actual proteolytic cleavage by the catalytic CLP protease core does not require ATP. Small substrate fragments (∼6–9 aa) are released from the CLP protease core through dynamic lateral pores, and once the substrate degradation is complete, the CLP chaperone–protease complex disassembles (11).

Recently, we took an *in vivo* CLP trapping approach in *Arabidopsis* that identified potential substrates and/or regulators interacting with *Arabidopsis* chloroplast CLPC1 (11), following strategies successfully used for substrates trapping of other AAA+ proteins in bacterial systems—reviewed in (5, 12). The *in vivo* trap was generated by expressing CLPC1 mutated in two critical glutamate residues in the two Walker B domains required for the hydrolysis of ATP and with a C-terminal STREPII affinity tag for purification (11). Affinity purification of the CLPC1-TRAP followed by tandem mass spectrometry (MS/MS) analysis resulted in a dozen proteins highly enriched compared with affinity-purified CLPC1 with a C-terminal STREPII affinity tag. These enriched proteins likely represent CLP protease substrates and/or new adaptors. Several of these trapped proteins overaccumulated in *CLP* mutants and/or were found as interactors of the adaptor CLPS1, supporting their functional relationship to CLP. The complete plastid protease core complex was strongly enriched in the CLPC1-TRAP eluates, providing the first robust support for CLPC and CLP core physical and functional interactions (11). This was the first *in vivo* trapping experiment with CLPC1. Although this study showed the proof of principle of chloroplast CLPC1 trapping, this study was carried out with a limited number of replicates and affinity-purified CLPC1 traps were analyzed with an older-generation Orbitrap mass spectrometer. A far more comprehensive *in vivo* trapping study should allow for a more robust dataset and potentially many additional candidate substrates, adaptors, or other regulators. This would be highly valuable also to make more informed choices as to which protein interactors to further pursue experimentally.

To obtain a more in-depth analysis of CLPC1 trapped proteins, we used the same genetic material as in (11) but carried out affinity purification and MS/MS analysis with a larger amount of leaf starting materials, more biological and technical replicates, and a far more sensitive and faster mass spectrometer. We also included an additional negative control line expressing an unrelated STREPII-tagged protease. Indeed, as described in this study, this greatly expanded the depth of analysis (many more proteins, better sequence coverage) and also allowed us to apply more robust protein quantification and enrichment analyses. The trapped proteins consisted of known plastid-localized proteins involved in various metabolic pathways and a set of proteins with different types of Domains of Unknown Function (DUF), as well as other uncharacterized proteins with UVR, Armadillo, or HugZ domains. Strikingly, several of these were of very low abundance as determined from inspection of public proteome resources (*e.g.*, PPDB, PeptideAtlas, SUBA) but were extremely enriched through the trapping approach. These proteins of unknown function could simply be substrates but should also be considered candidates for a regulatory role in CLP proteolysis, *e.g.*, as a modulator of CLPC chaperone or CLPPR protease activity, as an adaptor, coadaptor, or antiadaptor in substrate selection or perhaps supporting the priming and oligomerization of the CLPC chaperones. In such cases, these proteins could have evolved with the CLP system, and we therefore set out to search for signals of coevolution between these interactors and the different components of the CLP system at the amino acid level. This study will provide a comprehensive analysis for these DUF, UVR, HugZ proteins and their homologs based on (i) phylogenetic and Evolutionary Rate Covariation (ERC) analyses, (ii) an analysis of protein sequence coverage by experimental peptides, possible posttranslational modifications, and protein abundance in different parts of the plant based on our recently launched Arabidopsis PeptideAtlas build#1 (http://www.peptideatlas.org/builds/arabidopsis/), and (iii) mRNA-based coexpression networks using information from ATTEDII (https://atted.jp/). The coexpression and ERC analyses will be used to infer possible functional and/or physical relationships between the CLP machinery and these enriched proteins and their homologs.

## Results and discussion

**Figure 1:**
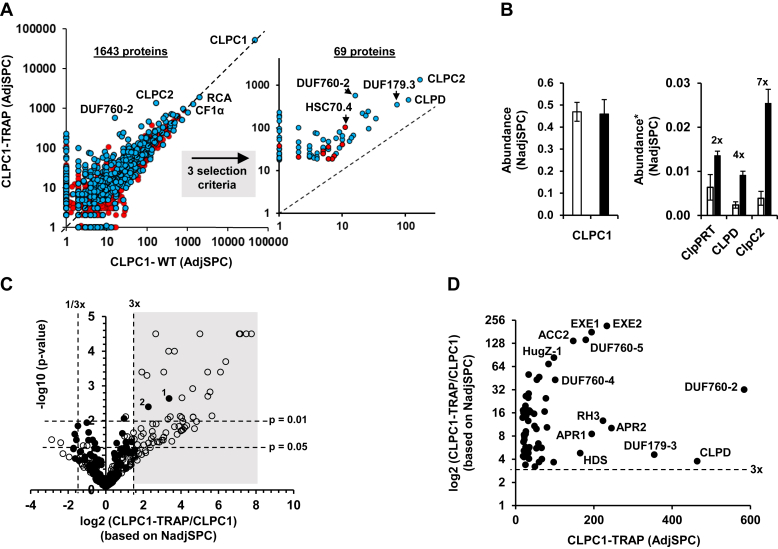
**Quantitative enrichment analysis of the eluates from affinity-purified CLPC1-WT-STREP and CLPC1-TRAP-STREP.***A*, scatter plot for the number of adjSPC in eluates of CLPC1-WT and CLPC1-TRAP for all 1643 proteins. The 575 proteins annotated as plastid proteins are marked in *blue* and the others marked in *red* (35%). Missing values for proteins not observed in one of the two genotypes are given the value one to allow for visualization on the log10 scale. The inset shows the subset of 69 proteins that remained after applying three selection criteria of the set of 1643 proteins. *B*, relative abundance of CLPC1 (*left panel*), CLPC2, CLPD, and the CLPRT subunits for the CLPC1-WT (*open bars*) and CLPC1-TRAP (*black filled bars*) affinity purifications based on NadjSPC. ∗Normalized to CLPC1. Standard deviations across the three biological replicates are indicated. The *left panel* shows that CLPC1 protein represented ∼45% of the total amount of proteins in the eluates. The *right panel* shows that the CLPPRT core complex, CLPD, and CLPC2 were, respectively, 2-, 4-, and 7-fold enriched in the CLPC1-TRAP eluates as compared with CLPC1-WT eluates. *C*, volcano plot for the 339 chloroplast proteins identified in the affinity eluates of the CLPC1-WT-STREPII and CLPC1-TRAP-STREPII lines based on NadjSPC (Table S1*B*). Proteins also identified in the CGEP-STREPII affinity eluates (negative control) are shown as filled circles. The *horizontal dashed lines* indicate *p*-values of 0.05 and 0.01 as indicated, whereas the *vertical dashed lines* indicate 3-fold enrichment in the CLPC1-TRAP (also indicated by the *gray* area) or CLPC1-WT eluates. Data point marked as 1 is CPN21 (AT5G20720), and datapoint marked as 2 is 4-hydroxy-3-methylbutyl diphosphate synthase (HDS) (AT5G60600). *D*, cross-correlation between the number of matched MS/MS spectra (adjSPC) for proteins in Table 1 and the log2 abundance ratio of proteins in CLPC1-TRAP-STREPII and CLPC1-WT-STREPII. Abbreviated protein names for selected proteins are indicated. For full names see Table 1.

**Figure 2:**
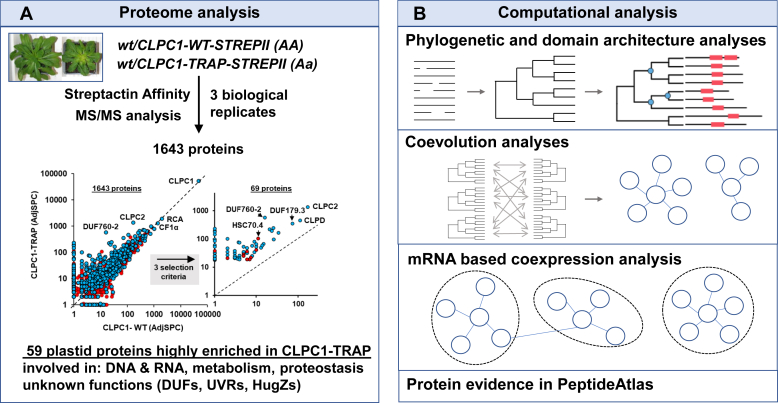
**Schematic overview of the proteome affinity analysis for CLPC1-WT and CLPC1-TRAP and four complementary bioinformatics analyses of selected proteins of interest.***A*, affinity protein enrichment using transgenic *Arabidopsis* plants expressing STREPII-tagged CLPC1-WT and CLPC1-TRAP with mutations in the Walker B motifs that block ATP hydrolysis. Proteins in the affinity eluates were identified and quantified by tandem mass spectrometry (MS/MS), and highly enriched proteins in the CLPC1-TRAP as compared with CLPC1-WT were selected for further analysis. The inset of the scatterplot was from Figure 1*A*. *B*, four complimentary bioinformatics analyses were carried out for a selected set of proteins of interest with unknown functions. These selected proteins are candidate adaptors for the CLP system. The phylogeny and domain analysis provide new evolutionary clues and information about possible redundancies. The ERC analysis aims to determine sign of coevolution of the proteins that make up the Clp system in chloroplasts and find coevolutionary signals between subunits of the Clp system and several of the enriched proteins. The mRNA-based coexpression analysis provides support for functional interactions between the Clp system and the enriched proteins. Mass spectrometry–based protein evidence across many public proteome datasets using the Arabidopsis PeptideAtlas database provided insight in the relative abundance and puts the enrichment in the CLPC1-TRAP in perspective. Because elucidation of Clp adaptor functions and even substrates can be so daunting, this comprehensive analysis of candidate adaptors and substrates allows for more rational choices of which proteins to select for functional studies and also help design the most promising experiments.

To screen for additional chloroplast CLPC1 chaperone interactors including potential substrates, adaptors, and antiadaptors, and to improve their protein sequence coverage and potential discovery of degrons, we carried out a comprehensive *in vivo* protein interaction screen with chloroplast CLPC1-WT and CLPC1-TRAP proteins expressed in *wildtype Arabidopsis*. Both transgenes are driven by a constitutive promotor, and each has a C-terminal STREPII tag that allows for efficient affinity enrichment (11). Prior transformation of the null *clpc1-1* line with the CLPC1-STREP transgene showed full complementation of the virescent phenotype and reduced biomass phenotype of *clpc1-1* (11). The two transgenes differ in that, in CLPC1-TRAP-STREPII, the critical glutamate residues in the two Walker B domains of CLPC1 required for hydrolysis of ATP (CLPC1-TRAP) are changed to alanines (E374A and E718A), whereas CLPC1-STREPII is unmodified. The transgenic plants were grown on soil, and rosettes were harvested in three batches per genotype before bolting; these different batches serve as biological replicates. Fig. S1 shows images of the plants just before harvest. The heterozygous CLPC1-TRAP-STREPII lines have reduced biomass, and phenotypes of the rosette leaves range from virescent in young leaves but wt-like green in mature, fully developed leaves (Fig. S1) The phenotype of the heterozygous CLPC1-TRAP line is less severe than the *clpc1-1* null mutant (11). The soluble leaf proteomes were isolated under nondenaturing conditions and applied to streptactin affinity purification. Affinity eluates were then subjected to SDS-PAGE, and gels were stained by Coomassie blue, followed by protein in-gel digestion with trypsin. Three biological replicates were analyzed. The resulting peptides for each biological replicate were extracted and analyzed by LC-MS/MS using triplicate runs that differed in acquisition parameters (technical replicates). Proteins were identified and quantified based on the number of matched MS/MS spectra using a well-established bioinformatics “pipeline” around the search engine Mascot (11) (and see Experimental procedures). Identified proteins were annotated for function and subcellular location using updated information from the Plant Proteome Database (PPDB). The CLPC affinity experiments identified 1643 proteins of which 575 were assigned to the plastid based on experimental support described in the literature (see PPDB) (Table S1*A*). The scatter plot in Figure 1*A* shows the number of spectral counts in CLPC1-WT and CLPC1-TRAP for all 1643 proteins; the 575 proteins that we have annotated as plastid proteins are marked up in blue. Figure 2*A* summarizes the proteomics workflow. These plastid proteins represented ∼72% of the protein biomass based on both adjusted Spectral Counts (adjSPC) and normalized adjSPC (NadjSPC). Previously, we also carried out a similar *in vivo* protein interaction analysis for transgenic plants expressing two different STREPII-tagged versions of the unrelated chloroplast glutamyl peptidase CGEP (13). As described (13), this did not identify any strong candidate interactors to CGEP, and this dataset therefore serves as an excellent negative control for nonspecific binding to the streptactin affinity columns and for abundant proteins. Proteins also identified in the CGEP-STREP affinity experiments are listed in Table S1.

### Enrichment of the complete chloroplast CLP system

CLPC1 was by far the most abundant protein in all replicates, averaging about 46% of all matched MS/MS spectra (Fig. 1, *A* and *B*). CLPC1 was observed in equal amounts in the CLPC1-WT and CLPC1-TRAP samples, with an average ratio of 0.98. This demonstrates that CLPC1 affinity enrichment was consistent and successful. The CLPC1 interactome included all known proteins of the chloroplast CLP system, including the adaptor CLPF, but excluding the adaptor CLPS1 (Table S1*A*). This lack of identification of CLPS1 by MS/MS is because it is a small protein (12 kDa) with relatively few suitable tryptic peptides (see also http://www.peptideatlas.org/builds/arabidopsis/); immunoblotting with CLPS1 specific serum previously showed that CLPS1 was enriched to the same extent as CLPF (11). All chloroplast CLPP (P1,3,4,5,6), CLPR (R1,2,3,4) core subunits as well as the peripheral CLPT1,2 core proteins (1, 2) were at least 2-fold enriched in CLPC1-TRAP as compared with CpC1-WT, whereas CLPF, CLPC2, and CLPD were 4- to 7-fold enriched (Fig. 1*B*). Together, this showed that the interaction between the CLP protease core and CLPC1 was stabilized by blocking ATP hydrolysis in CLPC1 through the Walker B mutations, supporting our previous findings (11).

### Enrichment analysis

We first used statistics to evaluate plastid proteins for potential enrichment in the CLPC1-TRAP or CLPC1-WT samples. We limited the plastid proteins to those with at least a total of 18 adjSPC across all experiments, resulting in 339 proteins. A volcano plot displays the log2 of CLPC1-TRAP/CLPC1-WT ratio and -log10 *p*-values based on the spectral counting data (Fig. 1*C*). Seventy-seven proteins were significantly (*p* < 0.01) different between CLPC1-TRAP and CLPC1-WT (Table S1*B* and Fig. 1*C*). Most of these (67) were enriched in the CLPC1-TRAP samples (upper right quadrant in Fig. 1*C*), and only 10 proteins were enriched in CLPC1-WT as compared with CLPC1-TRAP (upper left quadrant in Fig. 1*C*). Thirteen proteins were also observed in the CGEP affinity eluates; the negative control, however, only two of these, stromal CPN21 and HDS, were at least 3-fold enriched in the CLPC1-TRAP (Fig. 1*C*; area marked up in gray), indicating that a 3-fold enrichment was a strong criterium for specific trapping in the CLPC1-TRAP.

To obtain a stringent (conservative) set of proteins enriched in the CLPC1-TRAP eluates for further evaluation, we required at least 3-fold enrichment in CLPC1-TRAP compared with CLPC1-WT. We also required either two or three observations across the three biological CLPC1-TRAP replicates and a minimal threshold of 18 matched MS/MS for proteins identified in the CLPC1-TRAP samples (averaging two matched MS/MS spectra for the nine [biological + technical] replicates). This resulted in a set of 69 proteins (Table 1) of which 59 are plastid localized (Fig. 1*A*-inset). These 10 proteins not assigned to the plastid nearly all have a low number of SPC (between 26 and 51 across all experiments), with the exception of Hsc70-4 with 117 SPC. Five are observed only in two of the three bioreplicates. One of them (AT2G13440) is likely plastid localized, and the others have diverse functions and are unlikely to be located in plastids. This showed that our experiments and bioinformatics workflow (including selection criteria for enrichment) indeed mostly find plastid proteins, and the ones not in the plastid have low number of matched spectra. Most of these plastid proteins (52/59) were observed in all three biological CLPC1-TRAP replicates. Important, these 59 proteins were identified with at least three independent nonredundant peptides (irrespective of charge state or posttranslational modification) (Table S1). Of these 59 plastid proteins, 54 also showed statistical significance at *p* < 0.05 and the remaining five were significant at *p* < 0.1 (Table 1). Of the 17 proteins, 12 identified as trapped proteins in our previous study (11) are also part of this set of 59 enriched proteins, supporting their functional interaction with the CLP complex (Table 1). Just 2 of these 59 proteins, CPN21 and HDS (marked as #1 and #2 in Fig. 1*C*), were also observed in the CGEP-STREP experiments, and they could be nonspecific interactors with CLPC1 or perhaps also functionally interact with both CLPC1 and CGEP (see further below).

**Table 1:** Fifty-nine proteins that are enriched in the CLPC1-TRAP compared with CLPC1-WT^a^

Protein identifier	Montandon *et al.* (2019) enriched in ClpC1-TRAP^b^	Protein annotation	Function	Observed in # bio reps of TRAP (out of 3)	Total AdjSPC (all 18 exp.)	AdjSPC WT (all)	AdjSPC TRAP (all)	Average TRAP/WT (based on NadjSPC)^c^	*p*-value^d^
ATCG00190.1		rpoB RNA polymerase (PEP) beta	DNA–RNA	3	35	3	32	12.6	0.01500
ATCG00180.1		rpoC1 RNA polymerase (PEP) beta	DNA–RNA	3	41	3	38	16.4	0.00820
AT5G46580.1		pentatricopeptide repeat (PPR) protein SOT1	DNA–RNA	3	82	15	67	4.0	0.02370
AT5G26742.1		DEAD box RNA helicase (RH3) (EMB1138; globular stage)	DNA–RNA	3	244	21	223	12.6	0.00010
AT4G36390.1		tRNA/rRNA methyltransferase	DNA–RNA	3	29	2	27	14.9	0.01470
AT4G31210.1		DNA-directed topoisomerase—dually targeted mitochondria & plastid	DNA–RNA	3	30	4	26	9.2	0.02230
AT4G09730.1		DEAD box RNA helicase, RH39 (nara12) 23S rRNA processing	DNA–RNA	3	27	1	26	26.4	0.01710
AT3G48500.1		nucleoid protein (pTAC10)	DNA–RNA	3	54	6	48	10.8	0.00770
AT3G10270.1		DNA gyrase B1—dual targeted plastids and mitochondria	DNA–RNA	3	24	4	20	6.7	0.04090
AT3G04260.1		SAP domain–containing protein (pTAC3)	DNA–RNA	3	58	5	53	9.9	0.00910
AT3G02060.1		DEAD/DEAH box helicase	DNA–RNA	3	18	0	18	13.9	0.02500
AT2G39670.1		tRNA/rRNA methyltransferase	DNA–RNA	2	21	0	21	16.5	0.01910
AT1G74850.1		pentatricopeptide (PPR) repeat (pTAC2)	DNA–RNA	3	25	0	25	19.7	0.01400
AT1G30680.1		DNA primase-helicase (dual chloro-mito)	DNA–RNA	3	37	2	35	17.1	0.01060
AT1G02150.1		pentatricopeptide repeat (PPR) protein (6 or 7 repeats). Coexpresses with RNAse E/G At2g04270	DNA–RNA	3	123	26	97	3.7	0.01700
AT5G67030.1		zeaxanthin epoxidase (ZEP)	metabolism	3	62	13	49	3.2	0.04950
AT5G64840.1		ABC transporter family protein (ATGCN5)	metabolism	3	49	2	47	15.6	0.00750
AT5G60600.1		4-hydroxy-3-methylbutyl diphosphate synthase (HDS)	metabolism	3	199	34	165	4.8	0.00400
AT5G52920.1		pyruvate kinase-2 (typically homotetramer)	metabolism	3	31	5	26	5.7	0.04290
AT5G45930.1		Mg-protoporphyrin IX chelatase - CHLI-2	metabolism	3	24	3	21	8.3	0.03680
AT5G13110.1		glucose-6-phosphate dehydrogenase 2 (G6PD2)	metabolism	3	26	3	22	7.2	0.04170
AT4G30720.1		pigment defective 327 (PDE327) - oxidoreductase	metabolism	3	43	9	34	4.1	0.05010
AT4G22240.1		fibrillin 1b (FBN1b)	metabolism	3	32	0	32	25.1	0.00790
AT4G21990.1	8	5′-adenylylsulfate reductase-3 (APR3)	metabolism	3	81	4	77	24.7	0.00120
AT4G15560.1		1-deoxy-D-xylulose 5-phosphate synthase (DXS1)	metabolism	3	66	8	58	6.4	0.01250
AT4G11570.1	6	ARPP phosphatase cpFHy2 or PYRP2 (high in clpc1, clps1)	metabolism	3	84	0	84	68.9	0.00040
AT4G04610.1	8	5′-adenylylsulfate reductase-1 (APR1)	metabolism	3	218	24	194	8.5	0.00040
AT4G04020.1		fibrillin 1a (FBN1a)	metabolism	3	74	14	60	3.7	0.02860
AT3G44720.1		arogenate dehydratase 4 (ADT4)	metabolism	3	34	1	33	50.3	0.00730
AT3G21200.1		GluTR binding protein (GBP or PGR7)	metabolism	3	24	3	21	5.2	0.06020
AT3G10970.1	7	PYRP2-related Haloacid dehalogenase (HAD) hydrolase	metabolism	2	54	0	54	43.1	0.00200
AT3G07630.1		arogenate dehydratase 2 (ADT2)	metabolism	3	25	4	21	4.3	0.07420
AT2G44530.1		ribose-phosphate pyrophosphokinase	metabolism	3	38	5	33	4.8	0.04410
AT2G35390.1		ribose-phosphate pyrophosphokinase 1/phosphoribosyl diphosphate synthetase 1 (PRSI)	metabolism	3	28	3	25	8.5	0.03000
AT2G29630.1		thiamine biosynthesis (thiC family)	metabolism	3	40	5	35	5.7	0.02780
AT1G62180.1	8	5′-adenylylsulfate reductase-2 (APR2)	metabolism	3	270	26	245	10.2	0.00010
AT1G36180.1		acetyl-CoA carboxylase - ACC2	metabolism	3	147	0	147	137.4	0.00000
AT5G51110.1		Rubisco assembly factor 2 (RAF2)	proteostasis	2	33	8	25	3.4	0.09030
AT5G51070.1		CLPD	proteostasis	3	575	111	464	3.8	0.00040
AT5G42390.1		stromal processing peptidase (SPP)	proteostasis	2	63	10	53	5.6	0.02050
AT5G20720.1		CPN20	proteostasis	3	89	9	80	10.3	0.00230
AT4G25370.1		CLPT1	proteostasis	3	24	0	24	18.3	0.01590
AT4G12060.1		CLPT2	proteostasis	3	38	6	32	5.4	0.03320
AT3G48870.1		CLPC2	proteostasis	3	1527	170	1356	6.3	0.00000
AT2G44650.1	10	CPN10–1	proteostasis	3	19	0	19	17.3	0.01810
AT2G03390.1		CLPF (adaptor)	proteostasis	3	76	10	66	5.6	0.01250
AT1G35340.1		LON-domain protein 2 (LON-like2)	proteostasis	3	25	3	22	7.9	0.03690
AT5G66050.1		UVR4 (DUF151 and UVR domain)	unknown	3	60	0	60	46.7	0.00150
AT5G24060.1	16	HugZ-1	unknown	3	98	0	98	83.5	0.00020
AT4G33630.1		Executer 1 (EXE1)	unknown	3	194	0	194	178.5	0.00000
AT3G29240.1	3	DUF179–3	unknown	3	427	73	354	4.6	0.00050
AT3G17800.1	2	DUF760–5	unknown	3	180	1	179	142.2	0.00000
AT2G14910.1	5	DUF760–4	unknown	3	103	2	101	43.0	0.00040
AT1G75380.1		UVR2 (DUF151 and UVR domain)	unknown	2	34	1	33	28.4	0.01070
AT1G48450.1	1	DUF760–2	unknown	3	600	16	584	32.1	0.00000
AT1G32160.1		DUF760–1	unknown	2	23	4	19	4.4	0.08430
AT1G27510.1	9	Executer 2 (EXE2)	unknown	3	233	0	233	215.7	0.00000
AT1G23180.1		armadillo repeat protein (ARM)	unknown	3	78	4	74	16.6	0.00220
AT1G19660.1		UVR3 (DUF151 and UVR domain)	unknown	2	20	0	20	16.1	0.01990

a At least 3-fold ratio of CLPC1-TRAP/CLPC1-WT based NadjSPC. All proteins have at least three independent peptides (different aa sequences). All proteins are localized to the plastid.

b Montandon *et al.*, 2019 JPR. Table 2 - enriched in ClpC1-TRAP - rank (1–17; 1 is most enriched).

c trap/wt NadjSPC (input 1.10–5 for zero; this only happened for wt).

d *p*-Value (normalized to ClpC1) (based on GLEE pVal NadjSPC).

The relation between relative abundance in the CLPC1-WT and CLPC1-TRAP eluates and the relative enrichment in the CLPC1-TRAP for the 59 plastid proteins is shown in Figure 1*D*. This illustrates, *e.g.*, that DUF760-2 has a high relative abundance in the CLPC1-TRAP sample and is 32-fold enriched as compared with CLPC1-WT, whereas EXE1, EXE2, and DUF760-5 are >200-fold enriched and identified with ∼200 matched MS/MS spectra.

### Evaluation of CLPC1-TRAP enriched proteins

The functions of the enriched proteins in Table 1 can be assigned to four groups: (i) 15 proteins involved in DNA or RNA metabolism, (ii) 22 proteins directly or indirectly involved in chloroplast metabolism, (iii) 10 proteins involved in proteostasis, including chaperones (CPN10 and CPN21) and subunits of protease systems (CLPT1, CLPF, CLPC2, CLPD, SPP, and Lon-like2), and (iv) 12 proteins with specific domains (DUF760, DUF179, DUF151, UVR, HugZ, and ARM) but with mostly unknown functions. We will first briefly summarize the proteins for each of these four categories in the next sections, followed by an extensive analysis of DUF, UVR, HugZ, and ARM proteins, including phylogeny, mRNA-based coexpression, and protein identification across hundreds of experiments using the recent release of the Arabidopsis PeptideAtlas (14). This extensive analysis is summarized in Figure 2*B*.

### Enriched proteins involved in DNA and RNA metabolism

Most of the 15 proteins involved in DNA or RNA metabolism were previously found to be enriched in *Arabidopsis* chloroplast nucleoids (15); their homologs in maize were also nucleoid enriched (16). These 15 proteins include two subunits of the plastid-encoded RNA polymerase (PEP) complex, several PPR proteins (including pTAC2 (17, 18) and SOT1 (19, 20, 21)), three DEAD box RNA helicases two of which are involved in splicing (RH3 (22, 23), RH39 (24)), as well as two putative tRNA/rRNA methyltransferases that have not been described previously. Proteins involved with chloroplast DNA include a DNA topoisomerase, DNA gyrase B1 (25, 26), a DNA primase/helicase (27, 28) and pTAC3 (29) and pTAC10 (30). None of these proteins were observed in the CGEP-STREP affinity purification (the negative control), and the enrichment in the CLPC1-TRAP ranged from 3.7 to over 100, with between 18 to 223 matched MS/MS spectra for proteins in the CLPC1-TRAP (Table 1). Their enrichment suggests that these proteins are degraded by the CLP system, perhaps because most of the leaves (rosettes) were fully developed and therefore likely to have a lower demand for these proteins involved in DNA and RNA metabolism since plastid gene expression and translation are expected to be reduced when leaves are fully developed. The data do not tell us whether the CLPC1 chaperone directly interacts with these proteins (functioning in DNA/RNA metabolism) when they are attached to the nucleoid or otherwise located in the stroma.

### Enriched proteins involved in metabolism

Of interest, none of the trapped proteins involved in metabolism were involved in (high abundance) primary carbon metabolism (*e.g.*, Calvin–Benson cycle or starch metabolism), but instead they are involved in six other metabolic pathways, namely, fatty acid metabolism (ACC2 and pyruvate kinase), phenylalanine synthesis (arogenate dehydratase 2 and 4 (ADT2,4), 5′-adenylylsulfate reductases-1,2,3 (APR1,2,3) involved in sulfur metabolism, the methylerythritol phosphate pathway (DXS1 and HDS), the thiamin pathway (THIC (31, 32) and ARPP phosphatase PYRP2 (33) and a PYRP2 homolog), tetrapyrrole synthesis (GluTR binding protein GBP (34, 35) and Mg-protoporphyrin IX chelatase CHLI2 (36, 37)), and nucleotide metabolism (ribose-phosphate pyrophosphokinases). The family of APR proteins, as well as PYRP2 and its homolog, were also observed in our prior, smaller-scale CLPC1-TRAP analysis (11). GBP interacts with glutamyl t-RNA reductase (GluTR), the controlling enzyme in the synthesis of heme and chlorophyll. Binding of heme to GBP inhibits its interaction with the N-terminal regulatory domain of GluTR1, thus making GluTR1 accessible for recognition and degradation by the CLP protease system (34). Indeed, CLPS1, CLPC1, CLPF, and GBP all interact with the N terminus of GluTR (34, 38) and loss-of-function mutants of CLPR2 and CLPC1 showed increased GluTR stability, whereas absence of GBP results in decreased GluTR stability (35). Finally, fibrillins 1A and 1B were highly enriched in the CLPC1-TRAP. These fibrillins mostly function as components of plastoglobules and they respond to a wide range of abiotic stress conditions, but their molecular function is not known (39). The enriched proteins described above are candidate substrates for degradation by CLPPR protease and less likely to function as CLP substrate adaptors or regulators.

### Enriched proteins involved in chloroplast proteostasis

All known chloroplast CLP core subunits were enriched at least 2-fold in the CLPC1-TRAP, most likely due to stabilization of the interaction between the CLPC hexamer with the CLPPRT core complex (11). Stromal processing peptidase (SPP), responsible for cleaving all chloroplast transit peptides (40, 41), was 5-fold enriched in the CLPC1-TRAP; SPP levels were consistently several fold higher in various loss-of-function CLP mutants (42, 43) suggesting upregulation of SPP in response to proteostasis stress or alternatively that SPP is stabilized when CLP capacity is reduced. LON-domain protein 2 (LON-like2) was 7-fold enriched. LON-like2 is part of a small family with LON-like1 (AT1G19740), LON-like3 (AT1G75460), and LON-like4 (At2G25740). LON proteases are found in plant organelles (LON1–4 in *Arabidopsis*) and have an N-terminal LON domain, an AAA+ domain, and the catalytic LON domain (44). However, the LON-like family members (also named the iLON family) only have an N-terminal LON domain, and they are unlikely to have proteolytic activity themselves (2). Just recently, LON-like1 was suggested to somehow repress the activity of chloroplast thioredoxin y2, but the molecular mechanism is unknown (45). We detected, in addition to LON-like2, LON-like1 and LON-like3 in the CLPC eluates. LON-like1 was identified with 15 matched MS/MS spectra and a CLPC1-TRAP/CLPC1-WT ratio of 5.9, whereas LON-like3 was identified with 194 MS/MS spectra at a 2.1-fold abundance ratio (Table S1). Although neither of these LON-like proteins passed our thresholds for Table 1, they do appear to get trapped in CLPC1 either because they are CLP substrates or perhaps because they are involved in regulating aspects of CLP substrate selection and degradation. The Rubisco assembly factor 2 (RAF2) (46) identified with 33 MS/MS spectra was 3.4-fold enriched in the CLPC1-TRAP, but the significance level of enrichment was relatively low (*p* = 0.09). Finally, both the chaperone CPN20 and its cochaperonin CPN10-1 (46, 47) were >10-fold enriched in the CLPC1-TRAP (Table 1). Their enrichment could reflect not only their involvement of substrate unfolding and/or delivery but also their degradation. We previously observed and highlighted a strong enrichment of CPN20 in protein interactome analysis of CLPT1,2 (43). Interesting, a recent cryo-EM structure of the affinity-purified chloroplast CLPPR protease complex from the green algae *Chlamydomonas reinhardtii* showed that a heterotetramer of CPN11, CPN20, and CPN23 associated with one of the axial sides of the CLP core complex to form a stable 550-kDa complex (48). It was suggested that this cochaperone complex could play a role in coordinating protein folding and degradation in the *Chlamydomonas* chloroplast.

### Enriched proteins with unknown function, their domains, and phylogeny

The enrichment analysis also identified 12 proteins with unknown function (Table 1). These are proteins with Domain of Unknown Function (DUF) 179, DUF760, a UVR domain together with a DUF151 domain (UVR2, UVR3, UVR4) or without a DUF151 domain (EXE1, EXE2, UVR1), a Heme oxygenase HugZ-like domain, or several armadillo repeat (ARM) domains (Table 1). Six of these proteins were significantly enriched in our previous CLPC1-Trap study (11) (Table 1). Except for EXE1 and EXE2, involved in chloroplast singlet oxygen stress response (49, 50, 51), none of these proteins have been studied previously. None of these proteins or their homologs have known or predicted functions as metabolic enzymes, and therefore they are potential regulatory proteins in CLP proteolysis, including functions as CLP protease adaptors and antiadaptors. In the remainder of this study, we focus on this interesting set of CLPC1 interactors (as also summarized in Fig. 2*B*).

The enrichment analysis identified one protein with a DUF179 (AT3G29240), assigned DUF179-3 (Table 1). However, inspection of the original proteome dataset (Table S1) identified one additional DUF179 protein (AT1G32160–DUF179-1) identified with 217 matched MS/MS spectra and 1.3-fold enriched in the CLPC1-TRAP. Homology searches of the *Arabidopsis* genome identified one additional member of this family (AT1G48450–DUF179-2) (Table 2).

**Table 2:** Summary of the features the CLPC1-trapped proteins without known functions and their *Arabidopsis* homologs

Protein id	Abbreviated name	This study in Table 1 or prior study^a^^,^^b^	Curated location (PPDB)	Predicted location^c^	Total AdjSPC (this study)^d^	Average CLPC1-TRAP/CLPC1-WT^e^	Conclusion coevolution ERC (Fig. 5) (in bold, most pronounced)	Conclusions from mRNA coexpression (Figs. 6 and 7) (in bold, most pronounced)	PeptideAtlas # experiments (Fig. 8)	Conclusion for protein abundance and CLPC1 interaction and trapping
AT1G23180.1	ARM	Table 1	plastid	C	78	16.6	coevolution of ARM with EXE2 and with CLP core and CLPC1/2	in module enriched for plastid proteostasis	108	Abundant protein and enriched in trap
AT1G33780.1	DUF179-1		plastid stroma	C	217	1.3	coevolution with UVR2/3 and DUF760-1	some connectivity	90	Abundant interactor to ClpC1, independent of trapping
AT3G19780.1	DUF179-2		unknown	S	0	nd	Coevolution of DUF760-3, DUF760-7 and DUF179-2	poor connectivity	38	Moderately abundant, but not a ClpC1 interactor. Perhaps not located in the plastid
AT3G29240.1	DUF179-3	Table 1 (b3)	plastid stroma	C	427	4.6	coevolution with DUF179-2	module enriched for UBI/ATG degradation	35	Moderately abundant interactor, enriched in trap
AT5G52960.1	DUF3143	a,b13	plastid stroma	C	19	2.4		DUF760-2, DUF760-3 and DUF3143 showed many connections to the tight CLPPRT cluster	74	Abundant, but not a strong ClpC1 interactor
AT1G32160.1	DUF760-1 (clade 1)	Table 1	plastid	C	23	4.4	Coevolution with DUF179–1 and CLPT1/2	small module of UVR1, DUF760-1, DUF760-5, DUF760-7, DUF760-8. Direct edges between DUF760-1, DUF760-7, DUF760-8 and HUGZ-1	115	Abundant, but not a strong ClpC1 interactor, but enriched in trap
AT1G48450.1	DUF760-2 (clade 1)	Table 1 (b1)	plastid	C	600	32.1		DUF760-2, DUF760-3, and DUF3143 showed many connections to the tight CLPPRT cluster	18	Low abundance, highly enriched in trap
AT1G63610.1	DUF760-3 (clade 2)	b4	plastid	C	821	2.1	Coevolution of DUF760-3, DUF760-7 and DUF179-2	**DUF760-2, DUF760-3, and DUF3143 showed many connections to the tight CLPPRT cluster; direct edge with HugZ-3**	105	Abundant, ClpC1 interactor, not strongly dependent on trapping
AT2G14910.1	DUF760-4 (clade 2)	Table 1 (b5)	plastid	C	103	43.0		some connectivity	12	Low abundance, highly enriched in trap
AT3G07310.1	DUF760-5 (clade 3)		unknown	M	6	4.5		module of UVR1, DUF760-1, DUF760-5; direct edges with UVR1,3 and DUF760-8	3	Very low abundance, enriched in trap
AT3G17800.1	DUF760-6 (clade 1)	Table 1 (b2)	plastid	C	180	142.2		small module of CLPD and DUF760-6	36	Moderately abundant interactor, highly enriched in trap
AT5G14970.1	DUF760-7 (clade 2)		unknown	C	13	10.9	**Coevolution with DUF760-3, DUF179-2 and CLPD**	**small module of UVR1, DUF760-1, DUF760-5, DUF760-7, DUF760-8. Direct edges between DUF760-1, DUF760-7, DUF760-8, and UVR1**	2	Very low abundance, enriched in trap
AT5G48590.1	DUF760-8 (clade 3)		unknown	C	0	nd		small module of UVR1, DUF760-1, DUF760-5, DUF760-7, DUF760-8. Direct edges between DUF760-1, DUF760-7, DUF760-8 and HUGZ-1	0	protein not detected; pseudogene?
AT4G33630.1	EXE1	Table 1	thylakoid	C	194	178.5		some connectivity	84	Abundant interactor highly enriched in trap
AT1G27510.1	EXE2	Table 1 (b9)	thylakoid	C	233	215.7	Coevolution with ARM	direct edges with DUF760-2 and ClpX2	74	Abundant interactor highly enriched in trap
AT5G24060.1	HugZ-1	Table 1 (b16)	plastid	C	97.7	83.5	Coevolution with CLPP3,4,5 and EXE1	Direct edges between DUF760-1, DUF760-7, DUF760-8 and HUGZ-1. HugZ1- direct edge to CLPP3 and CLPP5	65	Abundant interactor to ClpC1, highly enriched in trap
AT3G49140.1	HugZ-2		plastid	C	11.3	10.9	Coevolution with CLPP3,4,5 and EXE1	direct edge with DUF760-3	108	Abundant, but not a strong ClpC1 interactor, but enriched in trap
AT3G59300.1	HugZ-3		plastid	C	9	9.4	**coevolution with CLPF and CLPP1, P3, R2, R4,T1/2, CLPS1, DUF760-3 and ARM**	poor connectiviy	6	Very low abundance, enriched in trap
AT3G09250.1	UVR1		plastid stroma	C	0	nd		**small module of UVR1, DUF760-1, DUF760-5, DUF760-7, DUF760-8; direct edges of UVR1 to DUF760-4,7, CLPP3,5**	59	Abundant, but not a ClpC1 interactor
AT1G75380.1	UVR2	Table 1	plastid	C	34	28.4	Coevolution with DUF179–1	**UBI/ATG degradation module of UVR2, UVR3 and DUF760-5. Direct edge between UVR2 and UVR3**	50	moderately abundant interactor, highly enriched in trap
AT1G19660.1	UVR3	Table 1	plastid	C	20	16.1	Coevolution with DUF179-1	**UBI/ATG degradation module of UVR2, UVR3 and DUF760-5. Direct edge between UVR2 and UVR3**	45	Moderately abundant interactor, enriched in trap
AT5G66050.1	UVR4	Table 1	plastid	C	60	46.7		poor connectivity	8	Low abundance, highly enriched in trap

a ClpS1 interactor, Nishimura *et al.* (2013).

b Trapped in ClpC1, Montandon *et al.* (2019); # indicates abundance rank.

c Predicted subcellular location by TargetP. C, chloroplast; M, mitochondria; S, secreted with signal peptide.

d Total AdjSPC, adjusted matched MS/MS spectra.

e Average TRAP/WT NadjSPC (input 1.10–5 for zero).

The enrichment analysis identified four proteins with a DUF760; we assigned these as DUF760-1,2,4,6. However, inspection of the original proteome dataset (Table S1) recognized three additional DUF760 proteins (DUF760-3,5,7), and searching the *Arabidopsis* genome revealed one additional member of this family (DUF760-8) (Table 2), which was, however, not observed in our CLPC1-trap experiments or in any other dataset in PPDB.

The enrichment analysis identified five proteins with a UVR domain, *i.e.*, EXE1, EXE2, UVR2, UVR3, and UVR4 (Table 1). In addition, the chaperones CLPC1, CLPC2 (but not CLPD), and CLPF also have UVR domains (7, 38). A search of the *Arabidopsis* genome revealed one additional protein with a UVR domain, assigned UVR1 (At3G09250) (Table 2).

The enrichment analysis identified one protein with a HugZ domain (IPR037119), assigned HugZ-1. Analysis of the original proteome dataset (Table S1) found two additional proteins with a HugZ domain (assigned HugZ-2 and HugZ-3)—these showed a 9- and 11-fold enrichment in the CLPC1-TRAP, respectively, but they did not pass our enrichment criteria owing to the relatively low number of matched MS/MS spectra (11 and 9, respectively) (Table 2).

Finally, the enrichment analysis identified one protein (AT1G23180) with four armadillo repeat (ARM) domains; we named it ARM (Table 2). The Armadillo repeat is a repetitive amino acid sequence of about 40 residues composed of a pair of alpha helices that form a hairpin structure (52). There are no close *Arabidopsis* homologs to ARM. It is interesting to note that ARM domains are frequently found in combination with U-box or F-box domains involved in proteasomal degradation. Examples are AT5G67340, AT2G44900 (ARMADILLO-1), AT3G60350 (ARABIDILLO-2) (53, 54, 55), as well as PUB4 E3 ligase (AT2G23140) involved in chloroplast degradation (56).

In our previous trapping study (11), we found another DUF domain protein to be enriched, DUF3143 (AT5G52960); this was 2.4-fold enriched in the CLPC1-TRAP in the current study and identified in all three biological replicates (Table S1). This protein was also identified as an interactor to CLPS1 (57). There are no *Arabidopsis* homologs of DUF3143.

**Figure 3:**
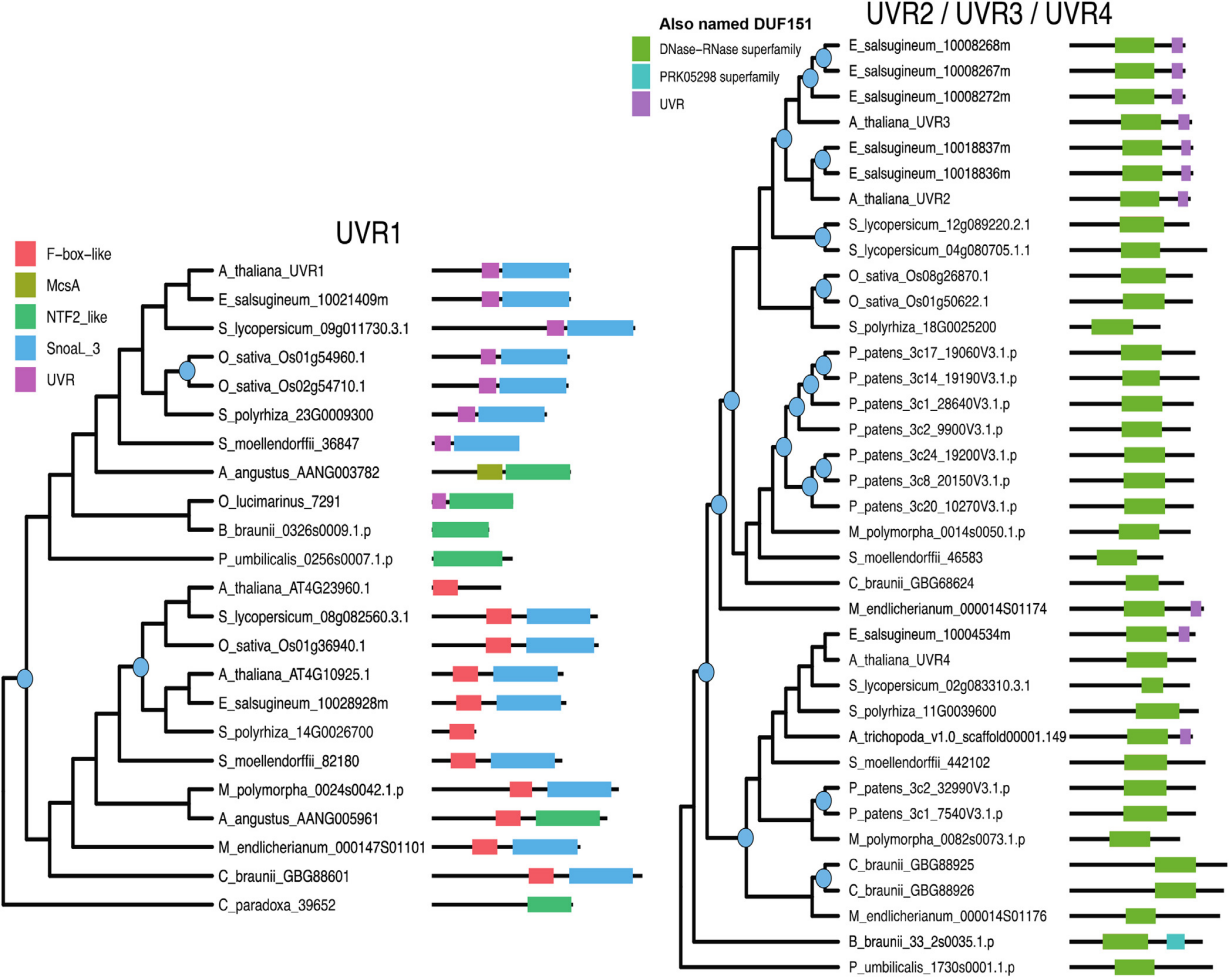
**Maximum likelihood trees and domain architecture diagrams for UVR1 and UVR2–4 protein families.** Inferred gene duplication events are indicated with blue circles at the corresponding node in the tree. Diagrams of domains were predicted by the NCBI conserved domain search tool CDsearch. Full species names and their lineage are: *Arabidopsis thaliana*, Angiosperms, Eudicots, Rosids, Brassicaceae; *Eutrema salsugineum*, Angiosperms, Eudicots, Rosids, Brassicaceae; *Solanum lycopersicum*, Angiosperm, Eudicots, Asterids; *Oryza sativa*, Angiosperms, Monocots, Poaceae; *Spirodela polyrhiza*, Angiosperms, Monocots; *Amborella trichopoda*, Angiosperms (earliest flowering plant); *Selaginella moellendorffii*, Lycophytes; *Marchantia polymorpha*, Bryophytes, Liverworts; *Physcomitrella patens*, Bryophytes, Mosses; *Anthoceros angustus*, Bryophytes, Hornwort; *Mesotaenium endlicherianum*, Charophytes, *Green algae*, Zygnemataceae (early-diverging); *Chara brauna*, Charophytes, *Green algae*; *Penium margaritaceum*, Charophytes, *Green algae*, Zygnemataceae; *Botryococcus braunii*, Chlorophyte, V, Trebouxiales; *Ostreococcus lucimarinus*, Chlorophyte, *Green algae*, Mamiellales; *Porphyridium purpureum*, Rhodophytes/*Red algae*; *Porphyra umbilicalis*, Rhodophytes/*Red algae*; *Cyanophora paradoxa*, Glaucophytes. Information about the functional domains and superfamily listed in the figure (see also Table S2): F-box-like, ∼50 amino acids long mediating protein–protein interactions in a variety of contexts, such as polyubiquitination, transcription elongation, centromere binding, and translational repression; McsA, domain found in the protein-arginine kinase activator protein McsA; NTF2-like, this superfamily (IPR032710) represents a domain covering the whole length of the nuclear transport factor 2 (NTF2). It has a β-α-β insertion after the main helix. Other proteins containing this domain include protein kinases, sucrose phosphatases, bacterial ring-hydroxylating dioxygenase beta subunit, protein NXF, and many other uncharacterized proteins. Snoal_3, SnoaL-like domain (IPR037401) is found in a large number of other sequences. SnoaL is a polyketide cyclase that adopts a distorted α-β barrel fold; Snoal_3 and NTF2 are overlapping superfamilies; UVR, this domain in UvrB can interact with the homologous domain in UvrC throughout a putative coiled coil structure. PRK05298 exonuclease ABC subunit UvrB. DNase-RNase superfamily (cl00553) with pfam02577 (DNase-RNase) and COG1259 (Bifunctional DNase/RNase), overlapping with DUF151, Bifunctional nuclease domain IPR003729, and BFN Bifunctional nuclease superfamily IPR036104; Arabidopsis AT4G10925 is an F-Box and Snoal protein.

**Figure 4:**
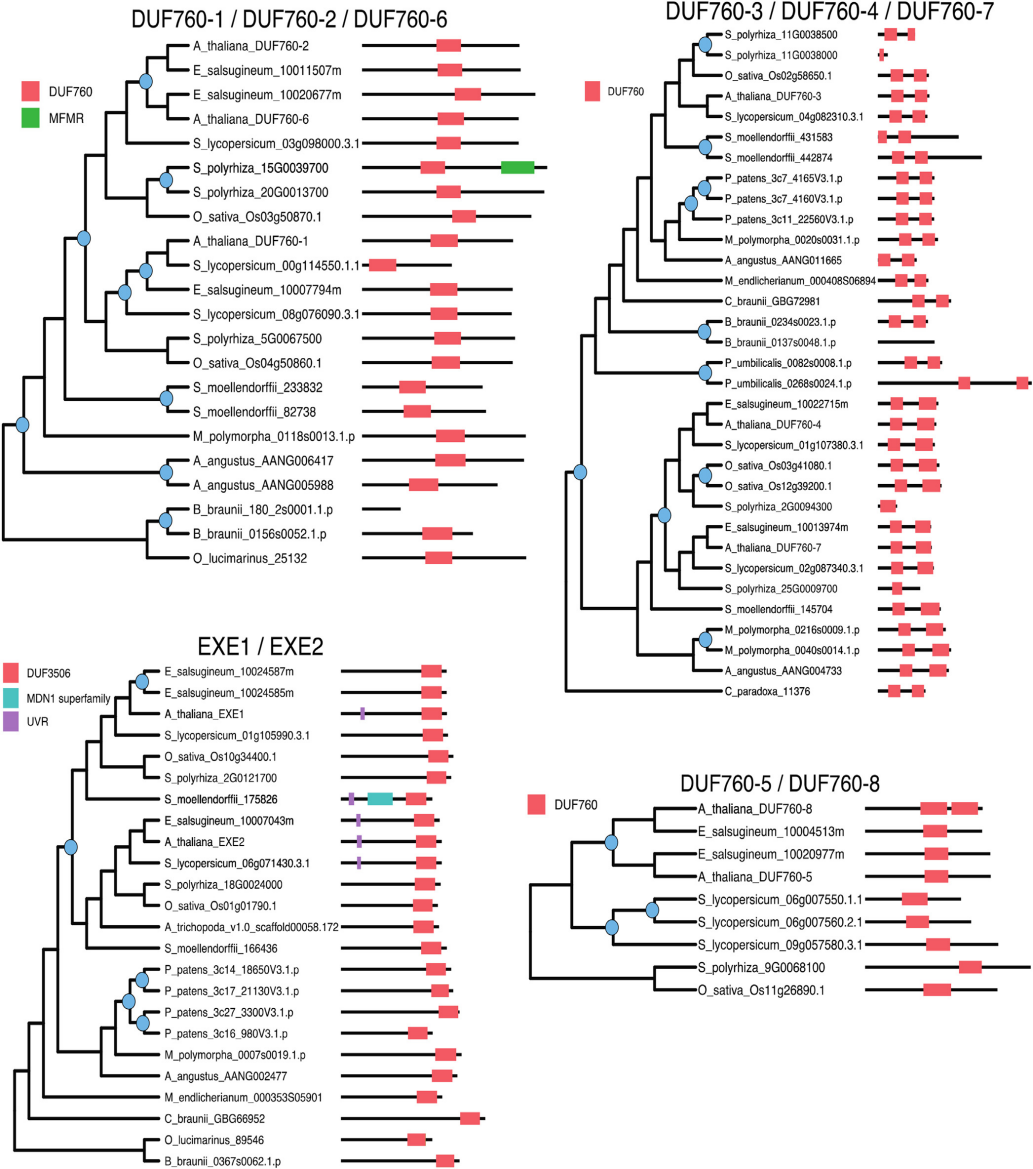
**Maximum likelihood trees and domain architecture diagrams for DUF760-1,2,6, DUF760-3,4,7, DUF760-5,8, and EXE1,2 protein families.** Inferred gene duplication events are indicated with *blue circles* at the corresponding node in the tree. Diagrams of domains were predicted by the NCBI conserved domain search tool CDsearch. For full species names and their lineage see legend to Figure 3. Information about the functional domains and superfamily listed in the figure (see also Table S2): DUF760 or pfam05542, protein of unknown function 760; MFMR G-box binding protein MFMR. Only one domain in this superfamily, pfam07777; MDN1 midasin AAA ATPase 1; UVR, pfam02151 is the only member in this superfamily.

**Figure 5:**
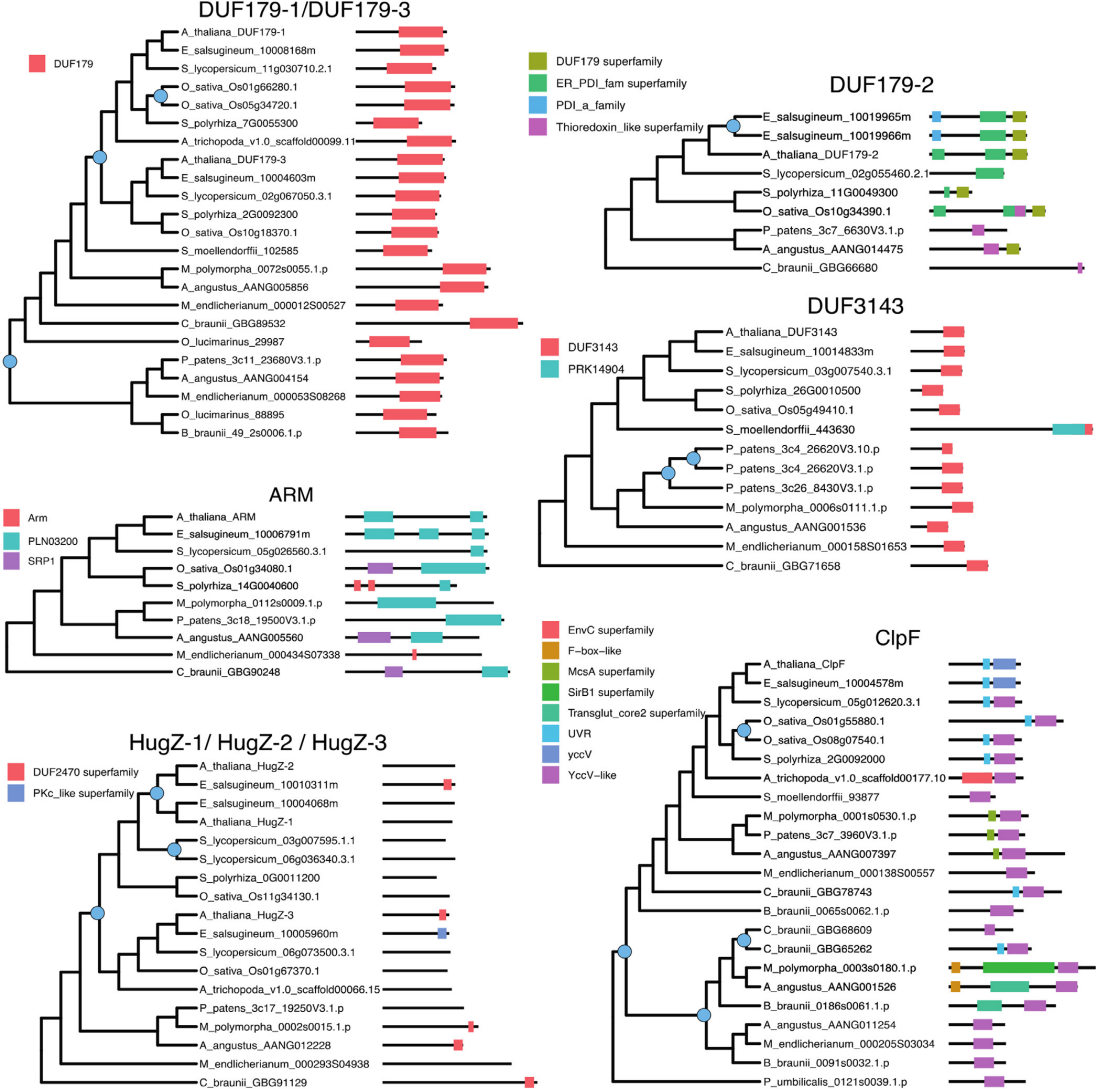
**Maximum likelihood trees and domain architecture diagrams for DUF179-1,3, DUF179-2, DUF3143, ARM, HugZ-1,2,3, and CLPF protein families.** Inferred gene duplication events are indicated with blue circles at the corresponding node in the tree. Diagrams of domains were predicted by the NCBI conserved domain search tool CDsearch. For full species names and their lineage see legend to Figure 3. Information about the functional domains and superfamily listed in the figure (see also Table S2): Arm, Armadillo/beta-catenin-like repeat of ∼40 amino acid repeat. Tandem repeats form super-helix of helices that is proposed to mediate interaction of beta-catenin with its ligands; PLN03200, cellulose synthase-interactive protein; SRP1, Karyopherin (importin) alpha; DUF2470, putative heme-iron utilization family; PKc_like superfamily, there are 60 domains in this superfamily. The protein kinase superfamily is mainly composed of the catalytic domains of serine/threonine-specific and tyrosine-specific protein kinase; DUF179, superfamily consists of pfam02622 (Uncharacterized ACR), COG1678 (AlgH), and PRK00228 (YqgE/AlgH family protein); ER_PDI_fam superfamily, protein disulfide isomerase; PDI_a_family, Protein Disulfide Oxidoreductases and Other Proteins with a Thioredoxin fold; Thioredoxin_like superfamily, Protein Disulfide Oxidoreductases and Other Proteins with a Thioredoxin fold; DUF3143, Protein of unknown function 3143, pfam11341 is the only member of this superfamily; PRK14904, 16S rRNA methyltransferase B; EnvC,superfamily, Septal ring factor EnvC, activator of murein hydrolases AmiA and AmiB; F-box-Like, ∼50 amino acids long mediating protein–protein interactions in a variety of contexts, such as polyubiquitination, transcription elongation, centromere binding, and translational repression; MscA, superfamily Protein-arginine kinase activator protein McsA; SirB1 superfamily, transglutaminaselike and TPR domain; Transglut_core2 superfamily, Transglutaminase-like superfamily has two domains: pfam13369 - Transglut_core2 and PRK10941 - tetratricopeptide repeat-containing protein; UVR, pfam02151 is the only member in this superfamily; yccV, domain in the small protein from *E. coli* YccV and its homologs in other Proteobacteria; YccV-like, superfamily has five domains pfam08755, TIGR02097, PRK14129 (HSPQ), smart00992, COG3785.

BLAST and functional domain searches against the *Arabidopsis* genome identified additional proteins with DUF179, DUF760, HugZ, and UVR domains resulting in a total set of 22 *Arabidopsis* proteins (Table 2). We searched for homologs of the 22 *Arabidopsis* proteins in 18 species across Archaeplastida with representatives from the glaucophytes, rhodophytes, chlorophytes, charophytes, bryophytes, lycophytes, and angiosperms and performed phylogenetic and conserved domain prediction analyses (Table S2 for more information). Based on this analysis, we mapped the 22 proteins to 10 gene families, and for comparison, we also included the CLPF protein family (Figure 3, Figure 4, Figure 5). With the exception of ARM (Fig. 5), all families underwent at least one gene duplication event within one or more species. Some show frequent duplications including at ancient nodes in the tree (*e.g.* UVR2/UVR3/UVR4, Fig. 3), whereas others show only recent lineage-specific duplications, meaning the gene remained single-copy throughout most of the tree (*e.g.* DUF3143, Fig. 5). Domain maps indicate that the level of conservation of domain architecture varies by gene family (see figure legends for details on the functional domains). Several genes exhibit conservation of one core domain paired with the occasional gain/loss of an additional domain (*e.g.* UVR2/UVR3/UVR4 (Fig. 3), EXE1/EXE2 (Fig. 4), CLPF (Fig. 5)). The UVR1 family presents a particularly interesting case of duplication and domain evolution (Fig. 3). Duplication occurred at an ancient point in Arachaeplastida evolution, and the two resulting paralogs diverged with one lineage acquiring a UVR domain and the other acquiring an F-box-like domain, exemplified by the two *Arabidopsis* F-box proteins (E3-ligases) AT4G23960 and AT4G10925 (neither have been studied) likely involved in substrate recognition for degradation by the proteosome. This pattern suggests neofunctionalization within proteostasis.

### Coevolution of CLP proteins and candidate CLP-interacting proteins

ERC is a method to reveal genes with a history of coevolution and/or shared evolutionary pressures, based on the concept that functionally related genes will experience correlated changes in rates of sequence evolution across a phylogeny (58, 59, 60, 61). Recently, we used ERC across angiosperms to demonstrate signatures of coevolution between plastid-encoded and nuclear-encoded proteins, in particular for proteins involved in plastid proteostasis (8, 59). For example, ERC analysis showed strong coevolution between the plastid-encoded CLPP1 and the nuclear-encoded CLPR and CLPP subunits of the CLP proteolytic core but the relationship between the nuclear-encoded proteins were not studied (8).

**Figure 6:**
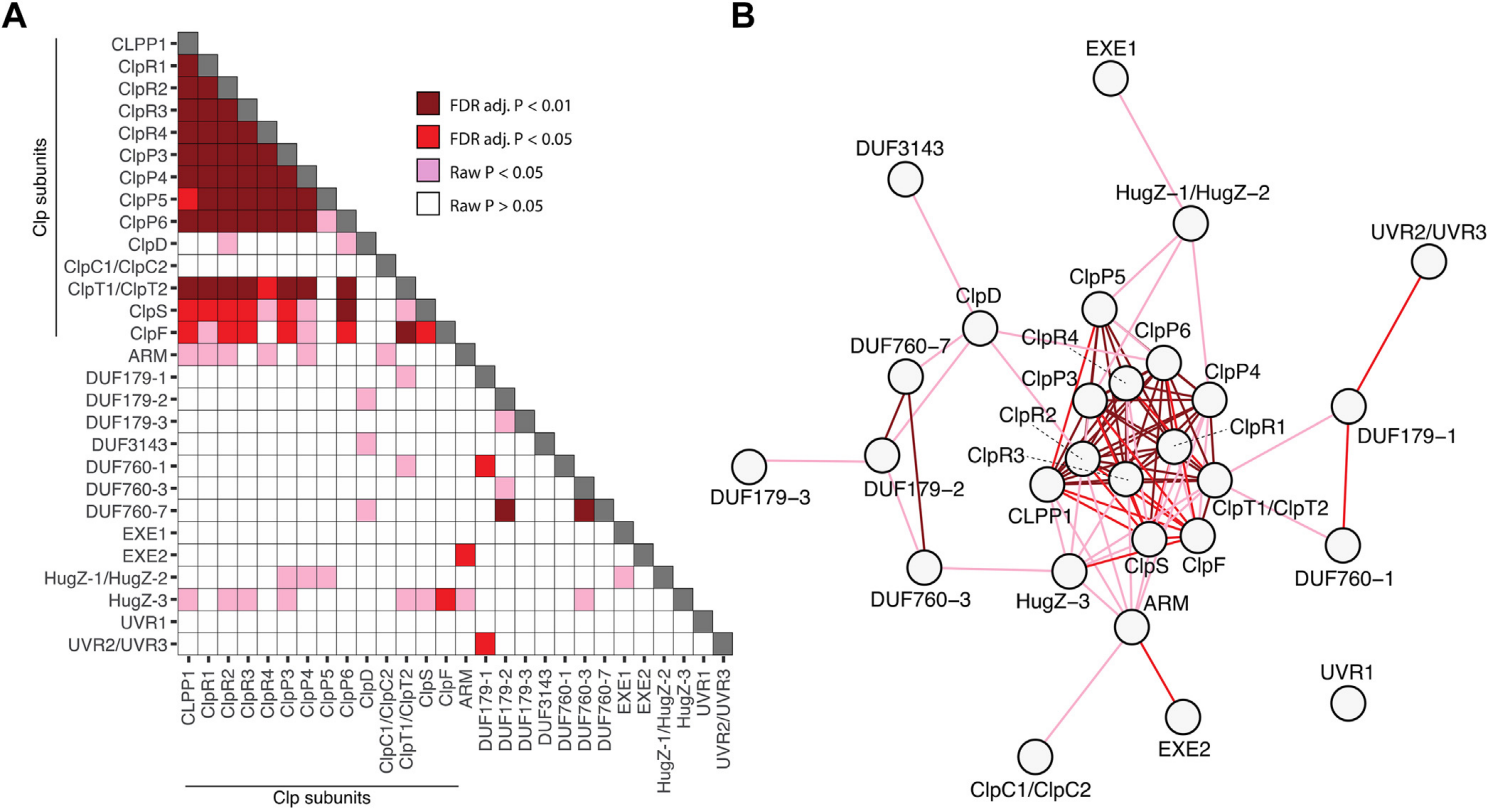
**Evolutionary rate covariation (ERC) between chloroplast CLP proteins and DUF179-1,3, DUF3143, DUF760-1,3,7, EXE1,2, HugZ-1,2,3, UVR1,2,3.***A*, results of ERC analyses between every pairwise combination of genes of subsets of genes. Pearson correlation *p*-values are indicated before (raw P) and after (FDR adj. P) multiple-test correction adjustment with the false discovery rate method. *B*, network diagram depicting ERC results. Connections of correlation using the same *colors* as in *A*.

We applied this ERC method to probe for coevolution between all subunits of the plastid CLP chaperone-protease system (CLPP1,3-6, CLPR1-4, CLPT1,2, CLPS1, CLPF, CLPC1,2 and CLPD) and the candidate interactors listed in Table 2 (Figs. 2*B* and 6). Figure 6*A* shows the full matrix with *p*-values, and Figure 6*B* displays the significant relationships as a network. This analysis showed strong coevolution between all subunit pairs within the CLPPR core and between CLPT1/T2 and the CLPPR core subunits, with the exception of CLPP5. This lack of coevolution for CLPP5 is surprising given that CLPP5 is essential for both structure and proteolytic function (62). There is strong ERC between the CLPS and CLPF adaptors and between CLPS1, CLPF, and members of the CLP core and CLPT1/T2 (Fig. 6). The exception is CLPP5, which does not show coevolution with CLPT1/T2, CLPF, or CLPS. On the other hand, the chaperones CLPC and CLPD show very little signature of coevolution with the CLP core. This lack of signature could reflect either false negatives or a true absence of selective pressure to coevolve, even while interacting (note that we did previously observe elevated CLPC rates in *Silene* species with rapid evolution in other CLP subunits (63)). For CLPD, this lack of signal likely results from a lack of power due to absence of the gene in many of the sampled species. Overall, the high degree of ERC within the CLP complex suggests coevolution between the CLPPR core, CLPT1,2, and CLPF and CLPS that reflects functional (but not necessarily physical) interactions within the CLP machinery.

We also found signs of ERC between CLP subunits and some of the CLP interactors (Fig. 6). In particular, HugZ-1/2, HugZ-3, and ARM show ERC signatures with several members of the plastid CLP system. For instance, HugZ-3 showed coevolution with CLPP1, P3, R2, R4 as well as CLPT1/2, CLPS1, and CLPF, suggesting that HugZ is functionally linked to the CLP system Interesting, a HugZ domain is also found in the C terminus of the *Arabidopsis* glutamyl-tRNA reductase (GluTR) binding protein (GBP) localized in chloroplasts. GluTR is important for the synthesis of 5-aminolevulinate, a precursor in heme and chlorophyll biosynthesis. Of importance, GBP plays a regulatory role in the stability of GluTR and protects the N terminus from being recruited by CLPS1 for degradation by the CLP system (34). This is quite a striking connection and suggests that the HugZ1/3 family could be directly involved in regulation of CLP substrate selection. Three DUF genes (DUF179-2, DUF3143, and DUF760-7) showed coevolution with the senescence and drought-induced CLPD chaperone, suggesting a functional connection. Finally, coevolutionary signatures were also found among pairs of candidate interactors. In particular, DUF760-7 showed coevolution with DUF179-2 and DUF760-3 (at adjusted *p*-value <0.05), whereas DUF179-1 showed a weaker coevolution signature with DUF760-1 and UVR2/3. These coevolutionary links provide a further incentive to study these interactors in more detail.

### Coexpression analysis of the CLP machinery and the trapped protein families

**Figure 7:**
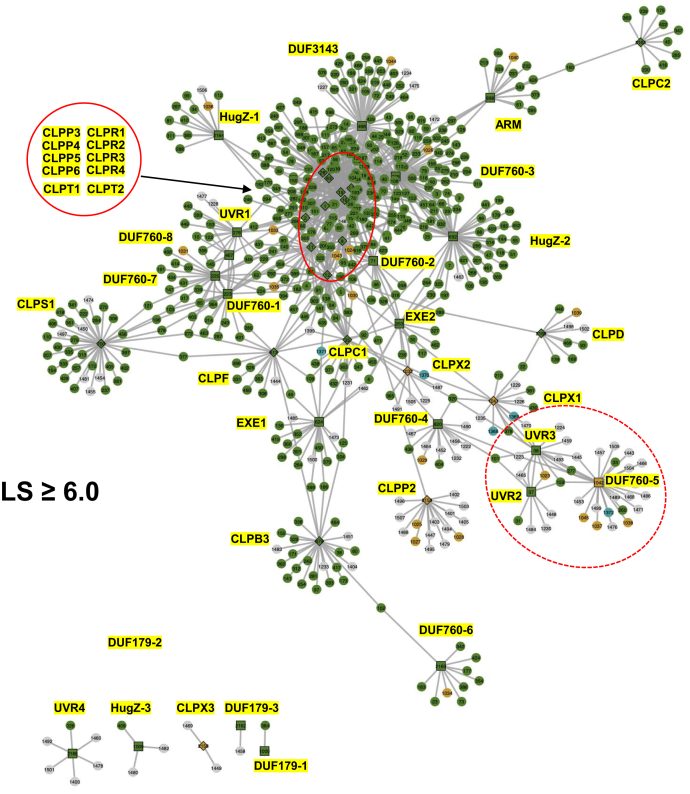
**mRNA-based coexpression network of trapped proteins, their *Arabidopsis* homologs, and the CLP system at LS ≥ 6.** The coexpression network was constructed using correlation data from ATTED-II based on both microarray and RNA-Seq experiments. One hundred genes with the highest coexpression values were downloaded for each of the 22 proteins in Table 2, as well as the complete nuclear-encoded chloroplast CLP system (15 proteins), the four mitochondrial CLP proteins (CLPP2, CLPX1-3), and the plastid unfoldase CLPB3, which does not directly physically interact with the CLP protease system. The network was generated after applying a minimal logit score (LS) of 6. The abbreviated names of the 44 baits are highlighted in *yellow*. A very tight coexpression cluster (*red ovals*) with all 10 nuclear-encoded members of the CLP protease core complex (CLPR1-4, CLPP3-6, CLPT1,2) is indicated.

A complementary tool to infer functional relationships between proteins is to study the correlation between mRNA expression levels across tissues or developmental stages in a single species, here *Arabidopsis* (Fig. 2*B*). To better understand the functional relationship of the trapped proteins and their homologs (Table 2) with the CLP machinery, we generated mRNA-based coexpression networks using correlation *Arabidopsis* data from ATTED-II based on both microarray and RNA-Seq experiments (64). We downloaded 100 genes with the highest coexpression values for each of the 22 proteins in Table 2, as well as the complete nuclear-encoded chloroplast CLP system (15 proteins), the four mitochondrial CLP proteins (CLPP2, CLPX1-3), and the plastid unfoldase CLPB3, which does not directly physically interact with the CLP protease system (Table S3*A*). This resulted in a set of 2157 nonredundant genes (Table S3). Coexpression was based on the logit score (LS), which is a monotonic transformation of the Mutual Rank index, with larger LS indicating stronger coexpression. We then constructed a coexpression network for the top 20 highest coexpressors of each of the 42 genes creating a network of 579 genes making 840 edges (1.45 edges/gene). We also generated coexpression networks based on two different minimal correlation thresholds for coexpression (LS ≥ 6 or 7) with 585 genes (1061 edges; 1.81 edges/gene) and 273 genes (414 edges; 1.52 edges/gene), respectively. Fig. S2 shows the three networks side by side, with bait names shown in yellow, plastid-localized gene products in green, mitochondrial localized gene products in orange, and gene products with unknown or other subcellular locations in gray. Each gene has the same identification number across the three networks (Table S3); 63%, 80%, and 85% of the proteins in the top20, LS ≥ 6, and LS ≥ 7 networks, respectively, were localized to the plastid. Figure 7 shows the LS ≥ 6 network.

In all three networks, the complete CLPPRT protease core complex formed a tight coexpression cluster, with CLPC1 and to a lesser degree CLPF, connected with multiple edges. CLPS1 was more distantly connected, with one shared coexpressor (Crumpled Leaf—AT5G51020) to CLPF (LS = 6.2/6.3). Interesting, DUF760-2, DUF760-3, and DUF3143 showed many connections to the tight CLPPRT cluster even at LS ≥ 7, suggesting that these three DUF proteins likely have a function closely associated with the plastid CLP system. At the highest stringency level (LS ≥7) (Fig. S2), only DUF760-1,2,3,7, DUF3143, HugZ-2, UVR1, EXE1, and EXE2 were part of the main network with the CLPPRT complex, CLPC1, CLPS1, and CLPF. Three proteins had no coexpressors at this highest stringency level (HugZ-3 and DUF179-1,2), and the other 11 proteins had between one (DUF179-3 and CLPX3) and 11 (CLPB3) coexpressors. The small DUF179 family only connected to the main network in the Top20 network (Fig. S2).

**Figure 8:**
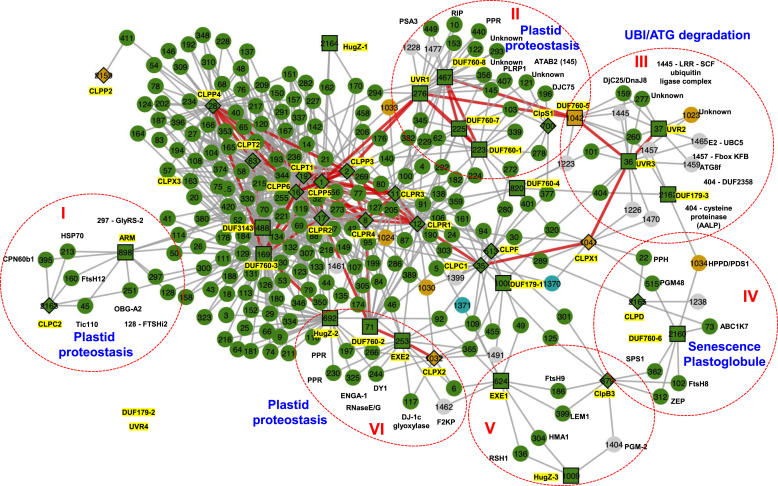
**Coexpression network of the combined top20 and LS ≥ 6 but including only those coexpressors with at least two edges.** The coexpression network was constructed using correlation data from ATTED-II based on both microarray and RNA-Seq experiments. One hundred genes with the highest coexpression values were downloaded for each of the 22 proteins in Table 2, as well as the complete nuclear-encoded chloroplast CLP system (15 proteins), the four mitochondrial CLP proteins (CLPP2, CLPX1-3), and the plastid unfoldase CLPB3, which does not directly physically interact with the CLP protease system. The network was then generated by including the top 20 coexpressors for each bait and the coexpressors for each bait with a minimal LS of 6. After combining these coexpressors, only those with at least two edges were kept. The abbreviated names of the 44 baits are highlighted in *yellow*. A very tight coexpression cluster with all 10 nuclear-encoded members of the CLP protease core complex CLPR1-4, CLPP3-6, CLPT1,2) is indicated. Modules are indicated with roman numbers and enriched functions are indicated in *blue fonts*. Direct edges between baits are represented with *thickened red lines*. All baits are numbered, and complete information about these coexpressors can be found in Table S3.

To more easily visualize the connectivity between CLP and trapped proteins, we generated a network of the combined top 20 and LS ≥ 6 coexpressors but including only those coexpressors with at least two edges (Fig. 8). This resulted in a dense network of 274 proteins and with 478 edges connected to CLP proteins and 311 edges connected to trapped proteins (average connectivity is 2.88 edges/protein); CLPX3, DUF179-2, and UVR4 were not part of this network. Ninety percent of the proteins are plastid localized. The direct edges between the baits (CLP and trapped proteins) are colored in red (see Fig. S3 for just the direct edge network). Again, the CLPPRT core formed a highly connected module, and DUF760-3 was an integral part of this module through direct edges to CLPR2, CLPP4, CLPP5, and CLPP6, suggesting a closely related functional role (Fig. 8). DUF760-2 was connected to this module through CLPR2 and CLPR4 (part of the R-ring), whereas UVR1 connected to CLPP3 and CLPP5 (part of the P-ring). UVR1, DUF760-1, DUF760-5, DUF760-7, and DUF760-8 formed a smaller module (module II), connected to the main module through edges of UVR1 to CLPP3 and CLPP5. UVR2 and UVR3 have direct edges and formed a small module (III) that included DUF179-3 and connecting to DUF760-5 and CLPX. Strikingly, several of the coexpressors in this module III encode for proteins involved in extraplastidic degradation through autophagy (ATG8f) and the UBI system. This is strongly contrasted to the dominant presence in most of the network for plastid proteins involved in various aspects of chloroplast biogenesis and proteostasis. CLPD and DUF760-6 form a small module (IV) connecting to DUF179-3, CLPX1, and CLPB3. Coexpressors in this module IV are mostly involved with senescence and plastoglobules, including the PG protease PGM48 (65) and atypical kinase ABC1K7 (66), as well as pheophytin pheophorbide hydrolase, a key enzyme in chlorophyll degradation (67).

### Protein observations in the Arabidopsis PeptideAtlas and comparison with CLPC1-TRAP and CLPC1-WT samples

To further evaluate the CLPC1-trapped proteins and their homologs, we took advantage of a new resource, the Arabidopsis PeptideAtlas (www.peptideatlas.org/builds/arabidopsis/) (14). Arabidopsis PeptideAtlas is based on publicly available mass spectrometry data from many published *Arabidopsis* proteome studies, collected through ProteomeXchange (http://www.proteomexchange.org/) and reanalyzed through a uniform processing and metadata annotation pipeline. In the first release, ∼40 million of ∼143 million MS/MS spectra acquired from a wide range of highly diverse samples from *Arabidopsis* (including leaves, flowers, roots, cell cultures, and subcellular fractions) were matched to the reference genome Araport11, identifying 17,858 uniquely identified proteins at the highest confidence level (canonical proteins) and 3543 lower confidence proteins. The raw MS datasets of the CLPC1 trapping experiment, as described above, as well as our previous CLPC1 trapping study (11) are also part of this atlas. In total there are 266 experiments in this peptideatlas.

**Figure 9:**
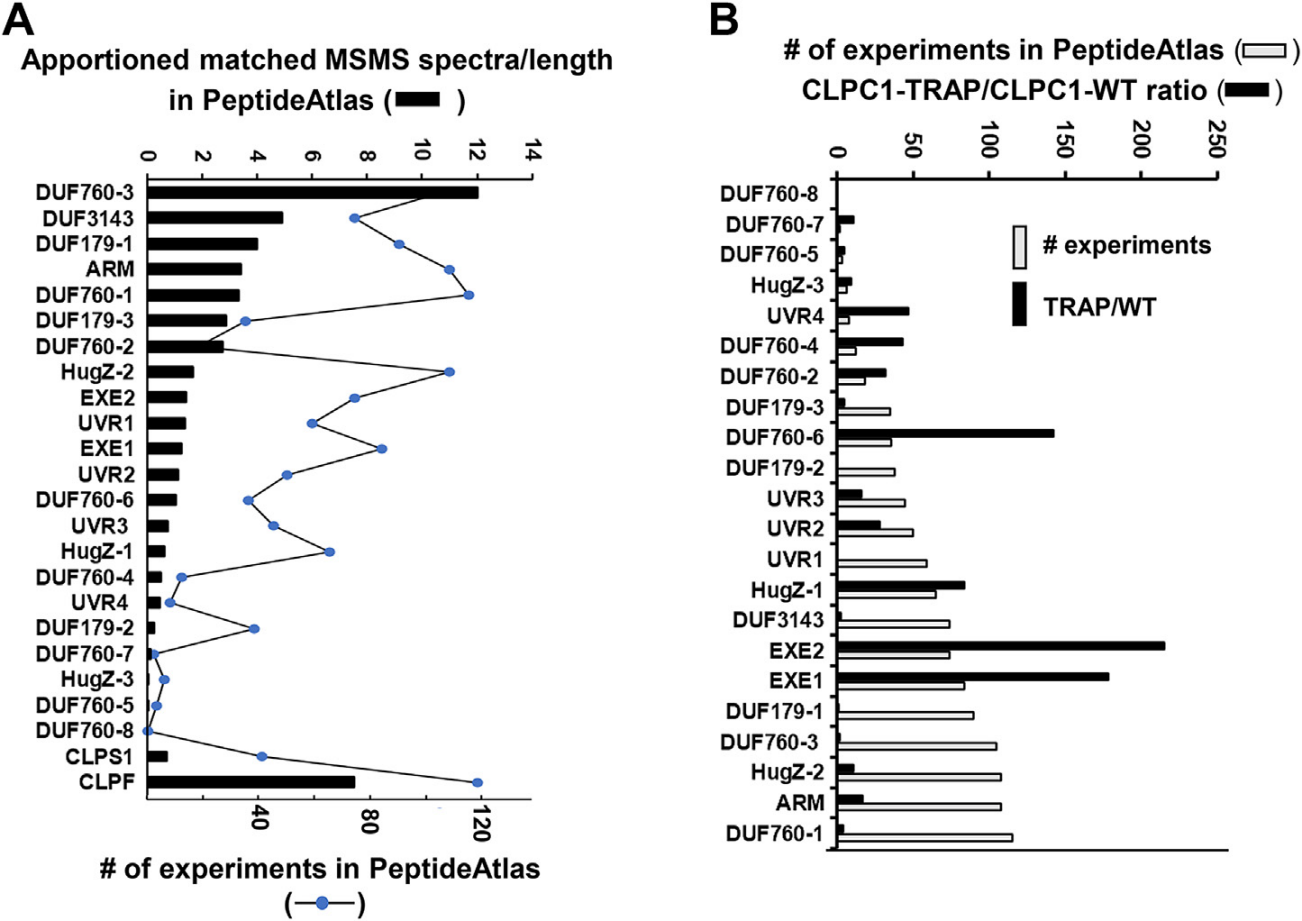
**Relative abundance and observations for proteins based on millions of MS/MS spectra in the Arabidopsis PeptideAtlas.** Arabidopsis PeptideAtlas is based on publicly available MS data for a wide range of highly diverse samples from *Arabidopsis* (including leaves, flowers, roots, cell cultures, and subcellular fractions) and reanalyzed through a uniform processing and metadata annotation pipeline. The MS data of the CLPC1 trapping experiments, as described above, as well as our previous CLPC1 trapping study (11) are part of this atlas. *A*, relative protein abundance in the current Arabidopsis PeptideAtlas based on apportioned matched spectra (PSMS) per length in number of amino acids for the 22 proteins in Table 2, and for CLPS1 and CLPF. *B*, number of experiments in the current Arabidopsis PeptideAtlas for which each of the 22 proteins in Table 2 and CLPS1 and CLPF were observed to provide a rough measure of abundance across the many sample types. Also shown is the relative enrichment in the CLPC1-TRAP compared with CLPC1-WT affinity experiments. This illustrates that the enrichment is independent of the general cellular protein abundance and underscores that the ClpC1-TRAP is highly selective.

**Figure 10:**
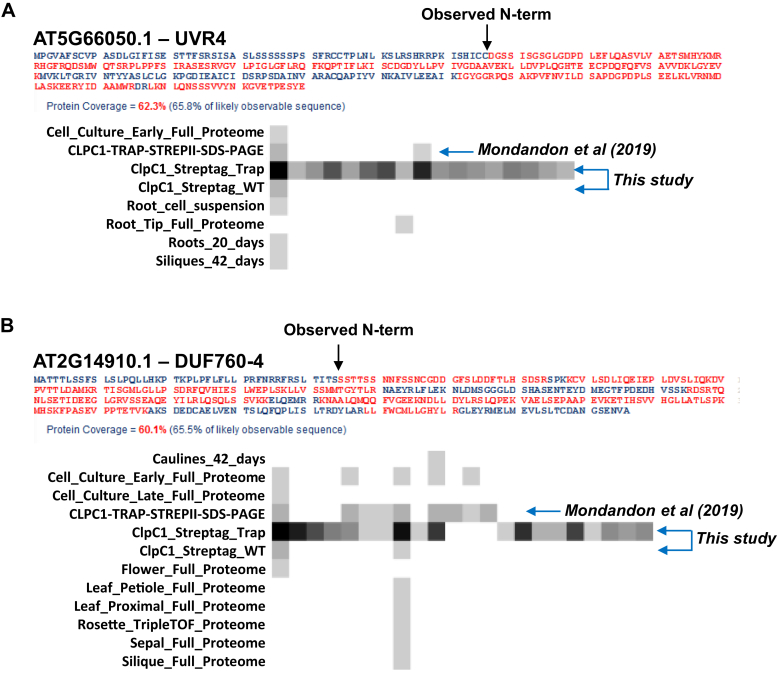
**Sequence coverage, relative abundance, and peptide observations for proteins based on millions of MS/MS spectra in the Arabidopsis PeptideAtlas.** Primary protein sequence and coverage by matched MS/MS spectra and detection of peptides across experiments in the PeptideAtlas for UVR4 (*A*) and DUF760-4 (*B*). Each square represents a unique peptide sequence. The gray scale reflects the number of PSMS for each peptide, with increasing darkness for increasing number of observations. It is highly likely that the N terminus of the protein accumulating in plastids was detected for both proteins because the most N-terminal peptide was not downstream of lysine or arginine residues and thus could not have been generated by the tryptic digest of the extracted proteome. For both proteins, by far the most PSMS were generated in the CLPC1-TRAP experiments in this study, with lower number of observations in the previous CLPC1 trapping study (11).

We collected information from PeptideAtlas for the 22 proteins including relative abundance (as matched number of spectra/protein length) across these very diverse datasets, overall protein sequence coverage by matched peptides, and the most N-terminal residue observed and evaluated in what datasets in PeptideAtlas these proteins were observed (*e.g.* tissue types, subcellular fractions) (summarized in Figs. S4–S10, Table S4, Figs. 9 and 10). Simplified information and a summary are provided in Table 2. All except one protein (DUF760-8) were identified at the canonical (most confident) level in PeptideAtlas. Some proteins were identified in more than 100 experiments (DUF760-1, DUF760-3, ARM, HugZ-2), whereas others were nearly exclusively identified in our CLPC1 affinity experiments (*e.g.*, UVR4, HugZ-3, DUF760-5, and DUF760-7), indicative of their low abundance and specific CLPC1 trapping (Fig. 9 and Table 2). For comparison, CLPS1 and CLPF were identified 41 and 118 times, respectively. The abundance of the canonical proteins in the current PeptideAtlas release (based on apportioned matched MS/MS spectra per protein length) ranges from 0.0018 to 1639 (the large subunit of Rubisco and CF1β of the thylakoid ATP synthase are the most abundant) (14), whereas the abundance of the 22 proteins (Table 2) ranged from 0.016 to 12.1 (Fig. 9*A*). DUF760-3 was by far the most abundant in this first PeptideAtlas release, whereas DUF760-5, DUF760-7, and HUGZ-3 were the least abundant and DUF760-8 was never observed (Fig. 9*A* and Table 2). For comparison, CLPS1, CLPF, and the average abundance of the CLPPRT subunits were 0.7, 7.6, and 25. We do note that these abundance numbers can vary greatly across experiments and tissue types, and therefore they do not directly correlate to abundance in one specific cell or tissue type; nevertheless, they provide a general measure of protein observability.

Figure 9*B* compares the CLPC1-TRAP/CLC1-WT ratio to the number of experiments in the PeptideAtlas with the proteins ordered based on increased number of experiments. This shows that the enrichment in the CLPC1-TRAP is not related to general abundance (or observability), *e.g.*, DUF760-6 is highly enriched in the CLPC1-TRAP but generally not that frequently observed in PeptideAtlas. Similarly, EXE1, EXE2, DUF179-1, and others are observed many times in the PeptideAtlas but only EXE1 and EXE2 are extremely enriched in the CLPC1-TRAP.

Figure 10 shows two examples (UVR4 and DUF760-4) of the primary sequence coverage and the peptide observations across experiments in the PeptideAtlas. In addition to our CLPC1 affinity experiments (>40 fold enriched in the CLPC1-TRAP compared with CLPC1-WT), UVR4 was detected mostly in nonphotosynthetic tissues (cell cultures, roots), whereas DUF760-4 was identified in a broader range of plant materials (leaves, flowers, and cell cultures) (Fig. 10*B*). However, for both proteins many more peptides were detected in the CLPC1-TRAP experiments showing that these proteins were truly highly enriched. All 21 observed proteins (Table 2) were identified with good sequence coverage in PeptideAtlas (32%–70%), whereas no peptides were identified in the N-terminal regions (see Figs. S4–S10 for all 21 proteins). The most N-terminal residue detected was at position 45; on average the most N-terminal residue was 69 aa from the N terminus supporting our prediction that (most of) these proteins have cleavable N-terminal chloroplast sorting sequences (chloroplast Transit Peptides or cTPs) (see Table 2). Moreover, for 11 proteins it was quite likely that the *bona fide* N terminus of the mature protein was detected because it was identified by a semi-tryptic peptide immediately downstream of a residue that was not K or R (hence not cleaved by trypsin) and with C-terminal K or R residues. Indeed, in most of these cases, the detected N terminus did fit the pattern of a cleaved cTP (*i.e.*, cleavage downstream of a cysteine, serine, or alanine) (see examples in Fig. 10). We did evaluate for possible plastid N-degrons (5, 68), and we observed three times a Leu (UVR3, HugZ-3, and DUF760-7) and once an Asp (UVR4) as the likely N-terminal residue. It was recently shown that N-terminal Leu is recognized by CLPS1 but that the following residue (the P2′ position) greatly affects the affinity, with Arg and also Gly enhancing the affinity but Leu, Ser, and Ala reducing affinity (68). Leu was followed by a Ser for HugZ-3 and DUF760-7 but Phe in case of UVR3. The significance of these N-terminal residues in the trapped samples remains to be determined.

## Conclusions

This study provides a comprehensive analysis of proteins that are copurified with CLPC1 chaperones in the *Arabidopsis* chloroplast, in particular when ATP hydrolysis of CLPC1 is impaired through Walker B mutations. In the absence of ATP hydrolysis, the interaction between CLPC1 and its substrates is stabilized (12). Since the main function of CLPC1 is the unfolding and delivery of substrates for degradation by the CLP protease complex, most of these interactors are likely protease substrates. However, it is quite likely that proteins that act in the regulation of CLPC1 hexamerization and activation could also be stabilized in their interactions with the CLPC1-TRAP. Finally, proteins that serve to select and deliver substrates (adaptors) to the CLPC1 chaperone may be unable to leave the CLPC1 chaperone if the substrate is unable to be unfolded and released into the CLP protease.

The CLPC1-TRAP plants do have pale green (virescent) young leaves, but these leaves green as they further develop and mature. The virescent phenotype must be accompanied with changes in the (chloroplast) proteome, and indeed, comparative proteomics of the homozygous *clpc1-1* null mutant previously observed a proteome phenotype (57). This *clpc1-1* null mutant has a much stronger phenotype (it is smaller and develops slower and its leaves are very pale) than the heterozygous CLPC1-TRAP line used for the current affinity enrichment. It is likely that proteins enriched in the CLPC1-TRAP line might also overaccumulate in the *clpc1-1* null line, and indeed, that was the case for several proteins, in particular EXE2 and DUF179.3.

This study identified 15 trapped proteins involved in DNA and RNA metabolism and 22 proteins involved in different chloroplast metabolic pathways; most of these are likely to be CLP protease substrates but protein half-life experiments in CLP-deficient backgrounds will be needed to investigate this further. Furthermore, another 10 proteins involved in chloroplast proteostasis were highly enriched in the CLPC1-TRAP; these include the CLPF adaptor, the CLPD chaperone, CLPT1 and CLPT2, as well as the CPN10/CPN20 cochaperone pair. Several of these proteins are direct components of the CLP chaperone-protease system (CLPF, CLPT1, CLPT2, CLPD). The >10-fold enrichment of cochaperone pair CPN10 and CPN20 is highly intriguing given the recent identification in the Chlamydomonas CLP core structure through cryo-EM (48); perhaps the CPN10/20 proteins also directly interact with the CLP protease core complex to regulate access to the catalytic chamber.

Most of this study focused on a set of proteins in families with unknown functions, *i.e.*, DUF179, DUF760, DUF151/UVR, DUF3143, HugZ, ARM, as well as EXE1 and EXE2. We identified 12 proteins in these families as being highly enriched in the CLPC1-TRAP, and analysis with BLAST and phylogeny identified another 10 members in these families, several of which were also enriched in the CLPC1 samples. Most (or perhaps all) of these 22 proteins localize to the chloroplast, suggesting that they specifically evolved to play a role in chloroplast metabolism or proteostasis. These proteins can perhaps serve as adaptors or in other regulatory functions in the Clp system and can also be substrates. Studies to determine possible regulatory functions such as CLP adaptor are difficult and often highly multiyear projects, as evidenced by the few examples published so far, in all cases for various types of bacteria. Just a few examples are (i) HSPQ in *Escherichia coli* which is now shown to be a regulator of Clp by inhibiting CLPS substrate selection but only if HSPQ is acetylated, thus HSPQ serves as an antiadaptor of CLPS (69); (ii) MecA in *Bacillus subtilis*, which not only acts as a substrate adaptor but also serves to functionally activate the CLPC hexamer (70), and (iii) the case of a tripartite adaptor system involving the adaptors CpdA, RcdA, and PopA in *Caulobacter crescentus* where RcdA can also be a substrate of the Clp protease system in dependence of its oligomeric state. It took several laboratories and many publications to begin to establish these regulatory functions. It is also important to note that several of these adaptors are themselves substrate for degradation by the Clp system (71). Because elucidation of Clp adaptor functions and even substrates can be so daunting, we carried out a comprehensive analysis of these 22 candidate adaptors and substrates through computational analysis (summarized in Fig. 2). We believe this will help to make more rational choices in selecting proteins for functional studies and also help design the most promising experiments.

We investigated for possible signals of coevolution with the CLP system and with each other and indeed several proteins; in particular the HugZ family members and ARM show signs of coevolution with the CLP system. Furthermore, specific members of the DUF760 and DUF179 families show strong coevolutionary signals, perhaps also indicative of protein–protein interactions between these members. To try and infer function, we used an in-depth mRNA-based coexpression network analysis. The complete set of CLPPRT proteins showed extremely tight coexpression consistent with a highly organized protein complex and further instilling confidence in the biological significance of the coexpression networks. Indeed, the coexpression networks suggest functional association of several of the proteins to specific functions or processes, such as the association of UVR2, UVR3, and DUF760-5 with members of the autophagy pathway and ubiquitination system, including several F-box proteins. These coexpression results will help to design experimental analysis for several of these proteins with unknown functions.

Finally, this study took advantage of the recent release of the Arabidopsis PeptideAtlas, which allowed a better understanding of the general abundance of the 22 proteins with unknown functions. This showed a wide range of abundance, and, importantly, showed that the CLPC1 trapping was highly specific as the enrichment in the CLPC1-TRAP showed no correlation with general abundance. Furthermore, the PeptideAtlas showed that all observed proteins accumulated without the first 50 to 70 amino acids, which is consistent with them having a cleavable chloroplast transit peptide for sorting from the cytoplasm (the site of protein translation for these nuclear-encoded proteins).

All together this comprehensive study provides a broad foundation to study the physiological role of the chloroplast CLP chaperone-protease system and discover molecular players and details of substrate delivery and regulation of CLP activity.

## Experimental procedures

### Plant material and plant growth

Homozygous *wt/CLPC1-WT-STREPII* and heterozygous *wt/CLPC1-TRAP-STREPII* transgenic lines used in this study are described in (11). Seeds were sown on agar plates with 50% Murashige and Skoog medium, 1% sucrose, and 20 mg/L BASTA. After 3 days dark stratification in the cold, these plates were transferred into to 10 h/14 h light/dark cycle at 100 μE m^−2^ s^−1^ to select transgenic lines carrying either transgene. After 10 days, surviving seedlings (100% for the homozygous *wt/CLPC1-WT-STREPII* line) were transferred to soil and grown under the same light/dark regime. Rosettes were harvested after 38 days just before bolting, divided in three separate batches per genotype, weighed, immediately frozen in liquid nitrogen, and stored at −80 °C. The different batches serve as biological replicates.

### Protein extraction and affinity purification

Batches of rosettes (10–14 g) were ground by pestle and mortar in liquid nitrogen to a fine powder and vortexed in 10 to 12 ml extraction medium (EM; 50 mM Hepes-KOH pH 8.0; 15% glycerol, 10 mM MgCl_2_, 75 mM NaCl, 0.32 mg avidin/ml EM, and 250 μg/ml pefablok serine protease inhibitor). The suspension was filtered through four layers of Miracloth (∼25 μm, Millipore), and larger particles were removed by centrifugation for 1.5 h at 28,000 rpm in a SW28 rotor at 4 °C. The supernatants were collected and aliquoted and either directly used for affinity purification on StrepTactinXT high-capacity affinity beads (# 2-4030-010 from IBA Life Sciences) or stored at −80 °C for later analysis. StrepTactin columns (0.5–1 ml) were prepared as in (72) and washed with 2 column volumes with EM without glycerol followed by equilibration with 2 column volumes of EM. Samples (0.5–1 column volumes) were loaded, the flow through was discarded, and columns were washed with 5 to 10 column volumes of elution medium (EM without avidin). STREPII tagged proteins were eluted in 3 column volumes of EM + 2.5 mM biotin (Biotin binds irreversible to Streptactin resin but is reversible with the newer-generation StrepTactinXT resin used here) and collected individually. The eluates were pooled and concentrated using Ultra-4 Centrifugal Filter Units with a 3-kDa cutoff by centrifugation for ∼16 h at 5000 rpm at 4 °C in a JS 13.1 rotor. The concentrates were aliquoted and stored at −80 °C for further proteome and MS/MS analysis.

### Proteomics and mass spectrometry

Affinity eluates of the transgenic lines expressing CLPC1-WT-STREP and CLPC1-TRAP-STREP were separated by SDS-PAGE on Biorad Criterion Tris-HCl precast gels (10.5%–14% acrylamide gradient) with three biological replicates. We refer to these eluates further as CLPC1-WT and CLPC1-TRAP. Each of the SDS-PAGE gel lanes were completely cut into consecutive gel slices (six per lane), followed by reduction, alkylation, and in-gel digestion with trypsin (73). The peptides resuspended in 15% formic acid were analyzed using a QExactive mass spectrometer equipped with a nanospray flex ion source and interfaced with a nanoLC system and autosampler (Dionex Ultimate 3000 Binary RSLCnano system). Peptide samples were automatically loaded on a guard column (C18 PepMap 100, 5 μm, 100 A; 300 μm i.d. × 1 mm; Thermo Scientific) *via* the autosampler followed by separation on a PepMap C18 reverse-phase nanocolumn (Inertsil ODS-3, 3 μm C18; 75 μm i.d. × 15 cm; Thermo Scientific) using 100-min gradients with 95% water, 5% ACN, 0.1% FA (solvent A) and 95% ACN, 5% water, 0.1% FA (solvent B) at a flow rate of 300 nl/min. Two blank samples were run after the six samples from each lane. The acquisition cycle consisted of a survey MS scan with a set mass range from 400 to 2000 *m/z* at the 70,000 resolving power followed by 10 data-dependent MS/MS scans with 2.0 *m/z* isolation window. Dynamic exclusion was used for 15 s. AGC target values were set at 1 × 10^6^ for the MS survey scans and maximum scan time 30 ms, and either 5.10^5^ or 5.10^4^ for MS/MS scans and maximum scan time 50 ms. Each sample was analyzed three times using different acquisition conditions (technical replicates) as follows: (i) 5.10^5^ MS/MS AGC and two internal washes with 95% B, (ii) 5.10^5^ MS/MS AGC and one internal wash with 95% B, and (iii) 5.10^4^ MS/MS AGC and one internal wash with 95% B.

### Data processing using MASCOT and our internal workflow

Peak lists in MGF format were generated from RAW files using Distiller software (version 2.7.1.0) in default mode (Matrix Science). MGF files were searched with MASCOT v2.4.0 against TAIR10 including a small set of typical contaminants and the decoy (71,148 sequences; 29,099,536 residues). Two parallel searches (Mascot *p*-value <0.01 for individual ion scores; precursor ion window 700–3500 Da) were carried out: (i) full tryptic (error tolerance 6 ppm for MS and 0.5 Da for MS/MS) with variable M-oxidation, Gln to pyro-Glu (N-termQ), N-term protein acetylation, W mono-, di-, or tri-oxidation and Fixed Cys-carbamido-methylation, two missed cleavages (in Mascot PR or PK does not count as missed cleavage), (ii) semi-tryptic (error tolerance 3 ppm and 0.5 Da for MS/MS) with variable M-oxidation, N-term acetylation, Gln to pyro-Glu (N-termQ), W-mono-, di-, or tri-oxidation, and fixed Cys-carbamido-methylation, two missed cleavages. W-oxidation was included based on the recent observations showing that a specific tryptophan residue in EXECUTER1 was oxidized (49). To ensure a final peptide false discovery rate below 1%, using a post-Mascot script, all search results were further filtered for minimum ion score of 33, but 35 for single peptide identifications. This resulted in a false discovery rate for proteins identified with two or more peptides of zero. Proteins identified by MS/MS spectra that were all shared with other proteins identified by unique peptides were discarded. Proteins could only be identified by the spectral counting method (SPC) with the full tryptic (6 ppm) search. The semi-tryptic search served to increase protein coverage and was combined with the full tryptic search results. Proteins were quantified by the spectral counting method (SPC) using full and semi-tryptic peptides search results. For quantification by spectral counting, each accession was scored for total spectral counts (SPC), unique SPC (uniquely matching to an accession), and adjusted SPC (73). The latter assigns shared peptides to accessions in proportion to their relative abundance using unique spectral counts for each accession as a basis. Proteins that shared more than 80% of their matched peptides with other proteins across the complete dataset were grouped into families quantified as groups with these homologs (73). We evaluated the samples for potential enrichment based on matched MS/MS adjusted spectra (adjSPC) normalized to the total number of adjSPC in each sample, resulting in NadjSPC. Alternatively, abundances of proteins within each lane were normalized based on adjSPC for CLPC proteins. Significance analysis for individual protein enrichment based on NadjSPC was done using the GLEE software developed in Phyton, and a stand-alone executable version of the software code was created (https://github.com/lponnala/glee-py) (A. Poliakov, L. Ponnala, P.D. Olinares, and K.J. van Wijk, unpublished data). GLEE was run in a Windows platform with a cubic polynomial equation fitting, adaptive binning, and 20,000 iterations for the estimation of variation. No normalization by protein length or peptide length was included. Volcano plots were generated in Excel.

### mRNA-based coexpression, networks, and functional enrichment

Coexpressed genes for the CLP and protein interactors families were downloaded (July 2020) from the plant coexpression database ATTED-II (http://atted.jp/) (64) using dataset Ath-u1. This dataset is a unified version of coexpression calculated by linear regression of both RNA-Seq and microarray coexpression data. The top 100 highest expressed genes based on the LS, a monotonic transformation of the Mutual Rank index, for each bait were used for detailed analysis. Larger LS indicates stronger coexpression, and LS = 0 indicates no coexpression. Protein function was based on an updated version of the MapMan annotation system integrated into the PPDB (http://ppdb.tc.cornell.edu/), and protein experimental or predicted subcellular location was obtained from PPDB. Proteins were assigned to plastid, mitochondria, peroxisome, or “other.”

### Gene duplication and domain architecture evolution

Complete sets of annotated protein-coding sequences for 18 species across Archaeplastida were obtained from published sources (Table S2) and processed to select only the primary gene model for each locus. Orthofinder (version 2.4.0) (74) was used to cluster gene families from the 18 species. Amino acid sequences were aligned using the L-INS-i algorithm in MAFFT (v7.407) (75). These alignments were manually inspected for assembly/annotation artifacts, and several sequences were found that appeared to be erroneously annotated as two neighboring partial proteins, each covering roughly half the length of the full-length protein. Such sequences were concatenated together to yield a single protein sequence for the given species. These curated sequences were used for domain analyses (see below). To prepare alignments for phylogenetic analyses, GBLOCKS (version 0.91b) (76) was used to trim poorly aligned regions. GBLOCK parameters b1, b2, and b5 were set such that conserved, flank, and gap positions were defined based on presence in at least 50% of sequences. RAxML (v8.2.12) (77) was used to infer maximum likelihood trees using the following command for each gene:

*raxmlHPC-PTHREADS-AVX -s <input file name> -n <output file name> -m PROTGAMMALG -p 12345 -x 12345 -# 100 -f a*. The -m argument indicated the model used (gamma distributed rate heterogeneity, empirical amino acid frequencies, and the LG substitution model). The -p and -x arguments provided a seed for parsimony search and bootstrapping, respectively. The -# argument indicates the number of bootstrap replicates. The -f a argument implements rapid bootstrap analyses and best scoring tree search. Gene-tree/species-tree reconciliation analyses were carried out using Notung (version 2.9) (78, 79). These analyses allowed comparison of each gene tree against a predefined species tree (80, 81) in order to identify gene duplication events, rearrange poorly supported nodes, and root trees in a manner that best matches the species tree. Default parameters were used for reconciliation and defined poorly supported relationships as those displaying <80% bootstrap support. The NCBI Conserved Domain search tool (CD-search) (82) was used to study the evolution of domain architecture of the selected gene families using the manually curated but untrimmed versions of the sequences (described above) using default parameters. Domain map figures were generated in R with the ggtree package (version 1.14.6) (83).

### Coevolution of CLP proteins and candidate CLP-interacting proteins

To search for evidence of coevolution between our proteins of interest, pairwise ERC analyses (58) was performed with 20 angiosperm species from a previously published dataset (59). *p*-Values were corrected for multiple tests using false discovery rate (84). The ERC network diagram was generated in R with igraph (85).

### *Arabidopsis* protein names and identifiers

CLPR1 - AT1G49970; CLPR2 - AT1G12410; CLPR3 - AT1G09130; CLPR4 - AT4G17040; CLPP3 - AT1G66670; CLPP4 - AT5G45390; CLPP5 - AT1G02560; CLPP6 - AT1G11750; CLPD AT5G51070; CLPS - AT1G68660; CLPC1 - AT5G50920; CLPC2 - AT3G48870; CLPT1 - AT4G25370; CLPT2 - AT4G12060; CLPF - AT2G03390; ARM - AT1G23180; DUF179-1 - AT1G33780; DUF179-2 - AT3G19780; DUF179-3 - AT3G29240; DUF3143 - AT5G52960; DUF760-1 - AT1G32160; DUF760-3 - AT1G63610; DUF760-7 - AT5G14970; EXE1 - AT4G33630; EXE2 - AT1G27510; DUF760-6 - AT3G17800; DUF760-4 - AT2G14910; DUF760 to 2 - AT1G48450; HUGZ-1- AT5G24060; HUGZ-2- AT3G49140.

## Data availability

The MS data have been deposited to the PRIDE Archive (http://www.ebi.ac.uk/pride/archive/) *via* the PRIDE partner repository and are available with the dataset identifier PXD017400. Matched posttranslational modifications as included in the Mascot searches, and limited information about MS-based identification results (peptide, ion score), as well as annotation of protein name, location, and function for the identified proteins can be found in the PPDB (http://ppdb.tc.cornell.edu/). The RAW files from PXD017400 were also processed as part of the Arabidopsis PeptideAtlas project and are available at http://www.peptideatlas.org/builds/arabidopsis/ (14). These PeptideAtlas data will be explored in this paper and compared with other *Arabidopsis* proteome datasets from other processed PXDs from ProteomeXchange.

## Supporting information

This article contains supporting information.

## Conflict of interest

The authors declare that they have no conflicts of interest with the contents of this article.
