# Supplementary material for: Proteomics, phylogenetics, and coexpression analyses indicate novel interactions in the plastid CLP chaperone-protease system
Source: J Biol Chem. 2022 Jan 20;298(3):101609. doi: 10.1016/j.jbc.2022.101609 (PMC8889267; doi:10.1016/j.jbc.2022.101609)
Supplement: Supplemental Figures S1–S10 [file mmc5.pdf]

Figure S1. Images of transgenic wt/CLPC1-STREPII (AA) and wt/CLPC1-TRAP-STREPII (Aa) plants just before harvesting. The bar (8 cm) shows the scale.

Figure S2. Three co-expression networks constructed using correlation data from ATTED-II based on both microarray and RNAseq experiments. 100 genes with the highest co-expression values were downloaded for each of the 22 proteins in Table 2, as well as the complete nuclear-encoded chloroplast CLP system (15 proteins), the four mitochondrial CLP proteins (CLPP2, CLPX1-3) and the plastid unfoldase CLPB3. Three networks were then generated using three different thresholds for selection of co-expressors for each bait, namely the top20 co-expressors, the co-expressors with a minimal LS of 6 or a minimal LS of 7 as indicated. The abbreviated names of the 44 baits are highlighted in yellow. A very tight co-expression cluster with all 10 nuclear-encoded members of the CLP protease core complex (CLPR1-4, CLPP3-6, CLPT1,2) was found in all three networks as indicated. All baits are numbered, and complete information about these co-expressors can be found in Table S3.

Figure S3. The direct mRNA co-expression edge network of the baits based on a network of the combined top 20 and LS ≥ 6 co-expressors.

Figure S4-10. PeptideAtlas sequence coverage and experiments for the 22 proteins in Table 2.

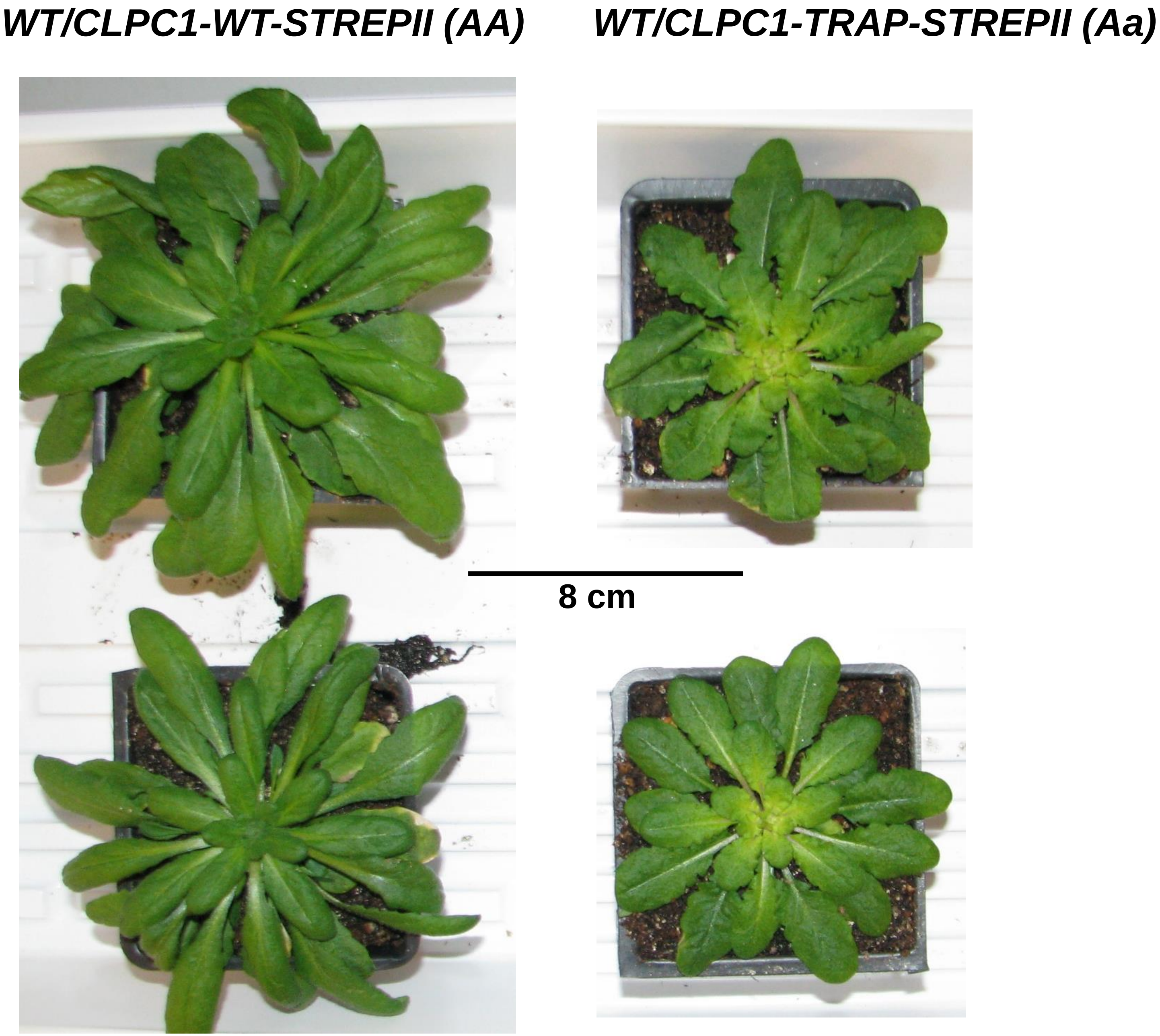

Figure S1

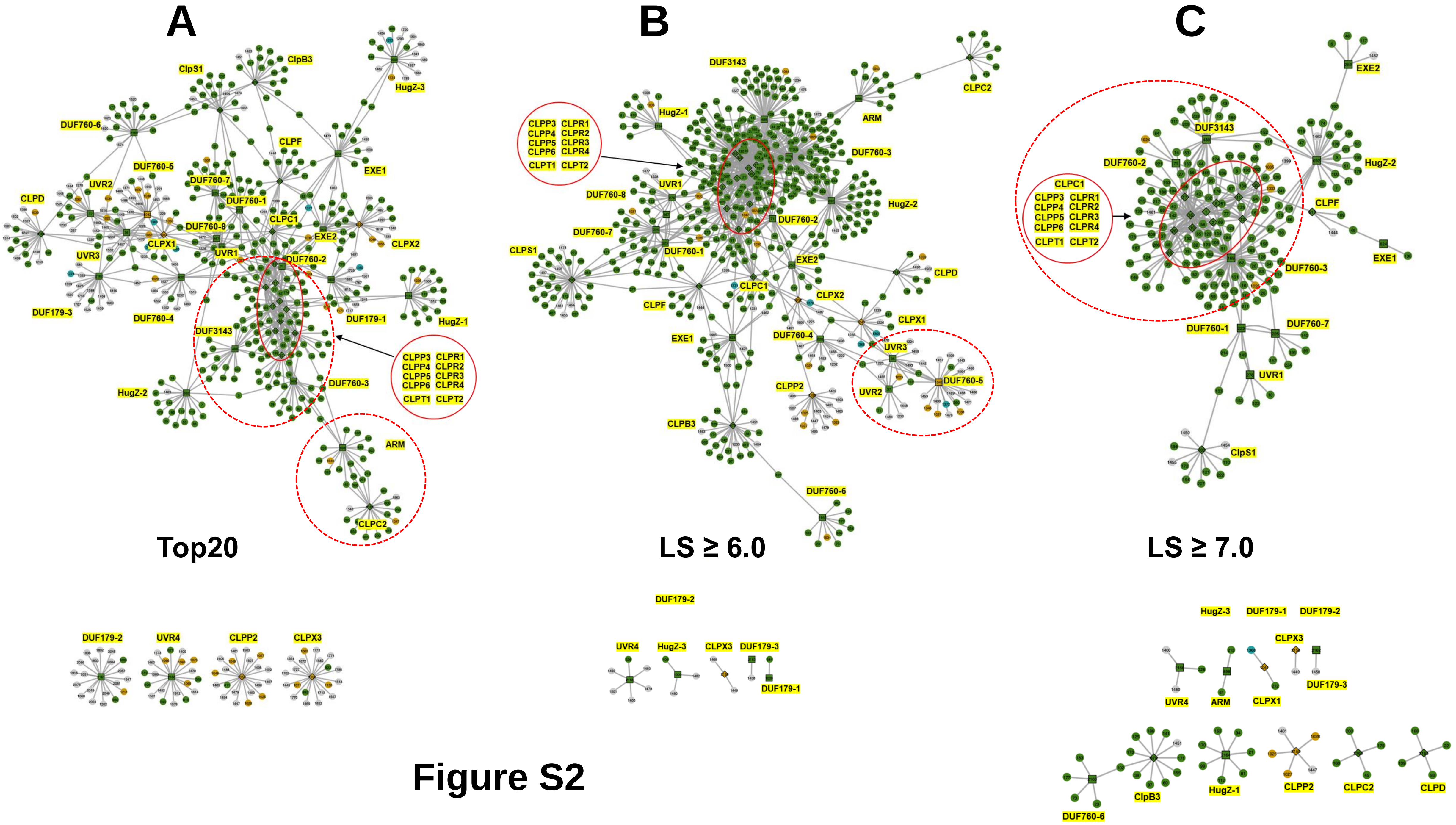
A
B
C
ClpS1
ClpB3
HugZ-3
DUF760-6
CLPF
EXE1
DUF760-5
UVR2
DUF760-7
CLPD
DUF760-1
CLPC1
DUF760-8
EXE2
CLPX2
CLPX1
UVR1
UVR3
DUF760-2
DUF179-3
DUF760-4
DUF179-1
HugZ-1
DUF3143
CLPP3 CLPR1
CLPP4 CLPR2
CLPP5 CLPR3
CLPP6 CLPR4
CLPT1 CLPT2
HugZ-2
DUF760-3
ARM
CLPC2
Top20
DUF179-2
UVR4
CLPP2
CLPX3
CLPS1
CLPB3
LS ≥ 6.0
CLPC1
LS ≥ 7.0
ClpS1
HugZ-3
DUF179-1
DUF179-2
DUF179-3
CLPX3

Figure S2

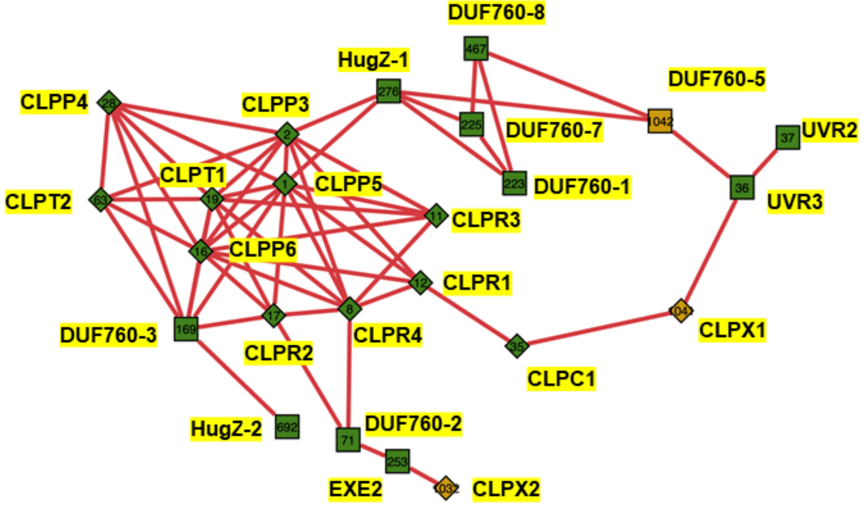

DUF760-8
HugZ-1
DUF760-5
CLPP4
CLPP3
DUF760-7
UVR2
CLPT1
CLPP5
DUF760-1
CLPT2
UVR3
CLPR3
CLPP6
CLPR1
CLPX1
DUF760-3
CLPR4
CLPR2
CLPC1
HugZ-2
DUF760-2
EXE2
CLPX2

Figure S3

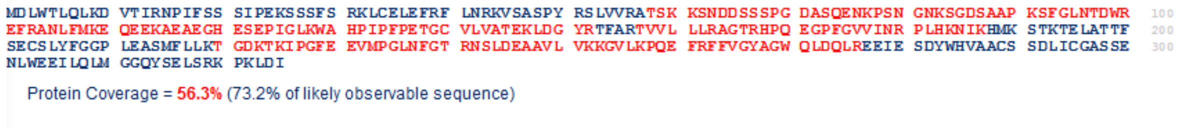

AT1G33780.1 – DUF179-1

start T68 after A67 -> **N-terminus**

25 peptides, 1294 observations.

90 experiments but by far the highest in CLPC1-Trap & CLPC1-WT

Very similar between WT and TRAP; <u>affinity does not require trapping</u>

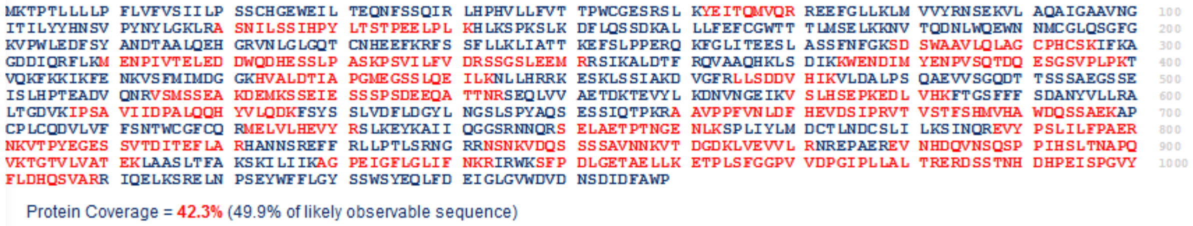

AT3G19780.1 - DUF179-2

start Y62 after K

30 peptides, 286 observations.

38 experiments – clearly highest in roots (several exp)

Not detected in CLPC1 experiments.

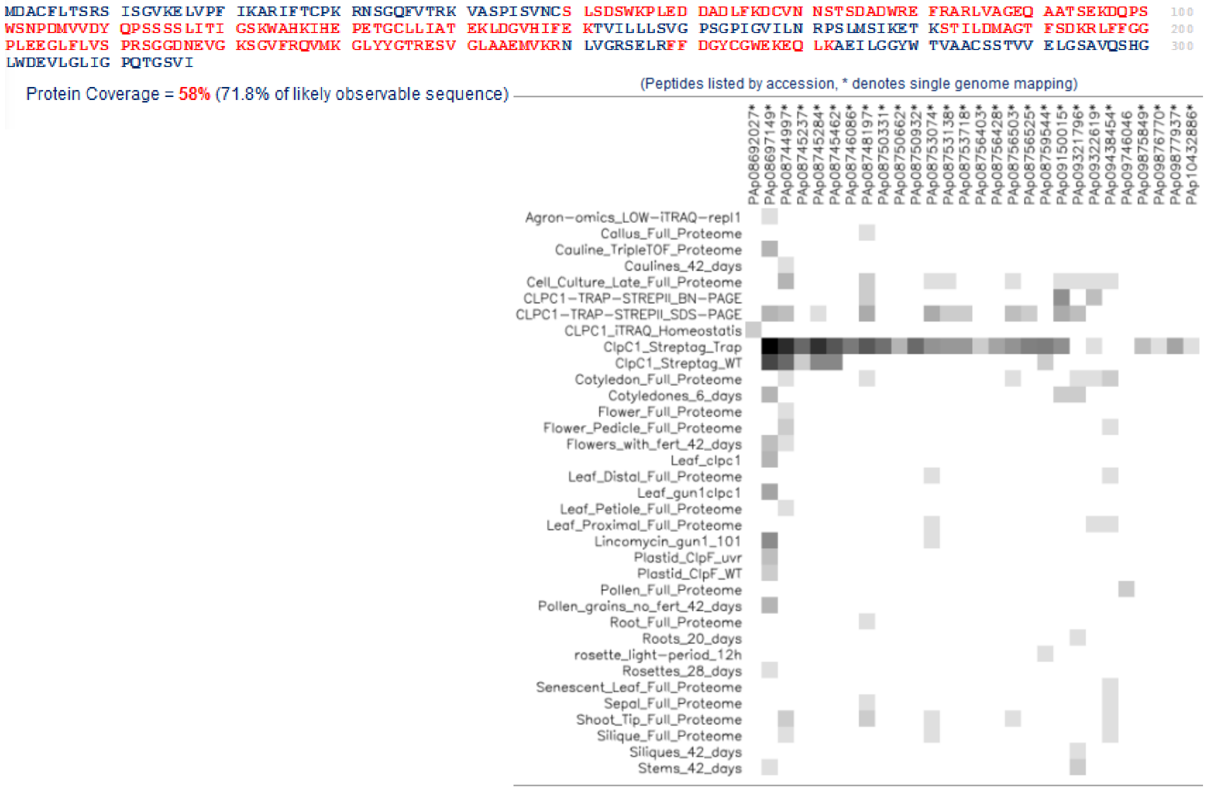

AT3G29240.1 - DUF179-3

start S50 after C -> **N-terminus**

28 peptides, 908 observations.

35 experiments but by far the highest in CLPC1-TRAP, followed by CLPC1-WT and Montandon data

Figure S4

MQAIGSSHGR RLCFNHYLSD SFSKFLSPSS SPSLLPQRCH SFCIPKLGSS STNENGRGRS VTVRASGDED SNENFAPLAP VELESPVGQL LEQILRTHPH 100
LLPVTVDEQL EKFAAESESR KADSSSTQDI LQKRISEVRD KERRKTLAEI IYCLVVHRFV EKGISMIPRI KPTSDPAGRI DLWPNQEEKL EVIHSADAFE 200
MIQSHLSSVL GDGPAVGPLS SIVQIGKIKL GKLYAASAMY GYFLRRVDQR YQLERTMNTL PKRPEKTRER FEEPSPPYPL WDPDSLIRIQ PEEYDPDEYA 300
IQRNEDESSS YGLRSYVTYL DSDTLQRYAT IRSKEAMTLI EKQTQALFGR PDIRILEDGK LDTSNDEVLS LSVSGLAMLV LEAVAFGSFL WDSESYVESK 400
YHFLKA

Protein Coverage = **50.7%** (58.1% of likely observable sequence)

AT1G32160.1 – DUF760-1

start A65 after R64

27 peptides, 1357 observations.

115 experiments; highest in leaf SEC, rosette & stems;
In both CLPC1-trap & CLPC1-WT – but not very abundant compared to other experiments

MDAVAANLFQ SPSLLRQTSE RLIFSTPNCP GFMRISSGPQ FRLRQNSSLK FSRPFQSGTT CVKSRRSFVV KASASGDAST ESIAPLQLKS PVGQFLSQIL 100
VSHPHLVPAA VEQQLEQLQI DRDAEEQSKD ASSVLGTDIV LYRRIAEVKE KERRRALEEI LYALVVQKFM DANVTLVPSI TSSSADPSGR VDTWPTLDGE 200
LERLHSPEVY EMIQNHLSII LKNRTDDLTA VAQISKLGVG QVYAASVMYG YFLKRIDQRF QLEKTMRILP GGSDEGETSI EQAGRDVERN FYEEAEETYQ 300
AVSSNQDVGS FVGGINASGG FSSDMKQSRL KTYVMSFDGE TLQRYATIRS RESVGIIEKH TEALFGRPEI VITPQGTIDS SKDEHIKISF KGLKRLVLEA 400
VTFGSFLWDV ESHVDSRYHF VLN

Protein Coverage = **70.6%** (78% of likely observable sequence)

Per-experiment expression for 40 most highly observed peptides

(Peptides listed by accession, * denotes single genome mapping)

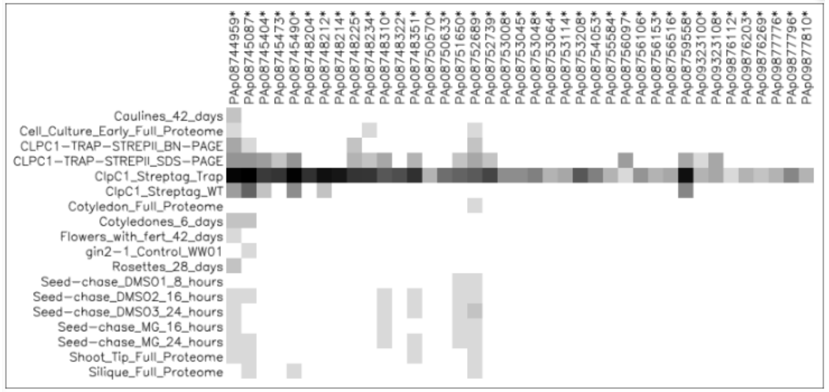

AT1G48450.1 – DUF760-2

start A72 after K71

51 peptides, 1176 observations

18 experiments and by far the most abundant in CLPC1-TRAP

Other experiments mostly various seed experiments

MCSLSMQFSL LQSPPSRPCP SFLANHEPKL STTSSSVTFP LKTNTWKCSG TGNLLVLRVK AYGSSSDSSA DSSTPPNGTR QPKSRRDILL EYVQNVKPEF 100
MEMFVKRAPK HVVEAMRQTV TNMIGTLPPQ FFAVTVTSVA ENLAQLMMSV LMTGYMFRNA QYRLELQQSL EQVALPEPRD QKGGDEDYAP GTQKNVSGEV 200
IRWNNVSGPE KIDAKKYIEL LEAEIEELNR QVGRKSANQQ NEILEYLKSL EPQNLKELTS TAGEDVAVAM NTFVKRLLAV SDPNQMKTNV TETSAADLAK 300
LLYWLMVVGY SIRNIEVRFD MERVLGTQPK LAELPPGEII

Protein Coverage = **66.4%** (77.3% of likely observable sequence)

AT1G63610.1 – DUF760.3

start A61 after K

55 peptides, 4082 observations.

105 experiments but by far the highest in CLPC1-Trap & CLPC1-WT quite similar for WT and TRAP; <u>affinity does not require trapping</u>

Figure S5

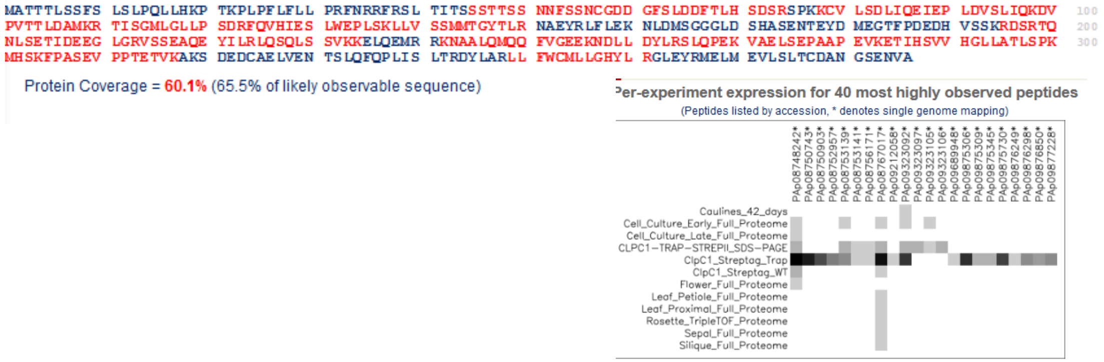

AT2G14910.1 – DUF760-4
start S45 after S **-> N-terminus**
22 peptides, 198 observations.
Only in 12 experiments, by far the highest in CLPC1-TRAP

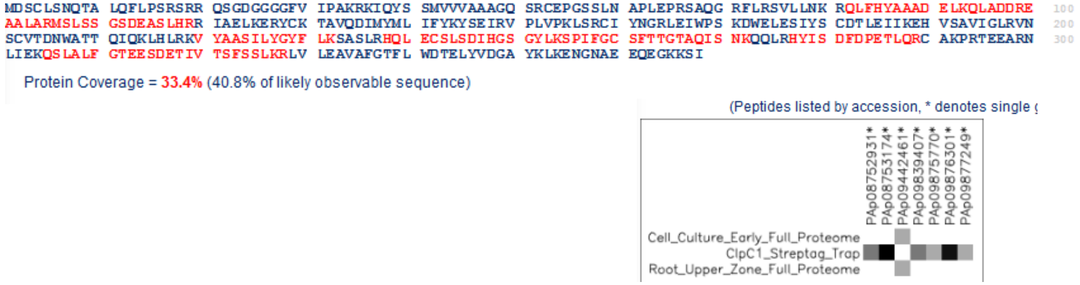

AT3G07310.1 - DUF760-5
start Q82 after R
7 peptides, 21 observations.
Only in 3 experiments (CLPC1-Trap, cell culture, root-upper zone)
By far the highest in CLPC1-TRAP

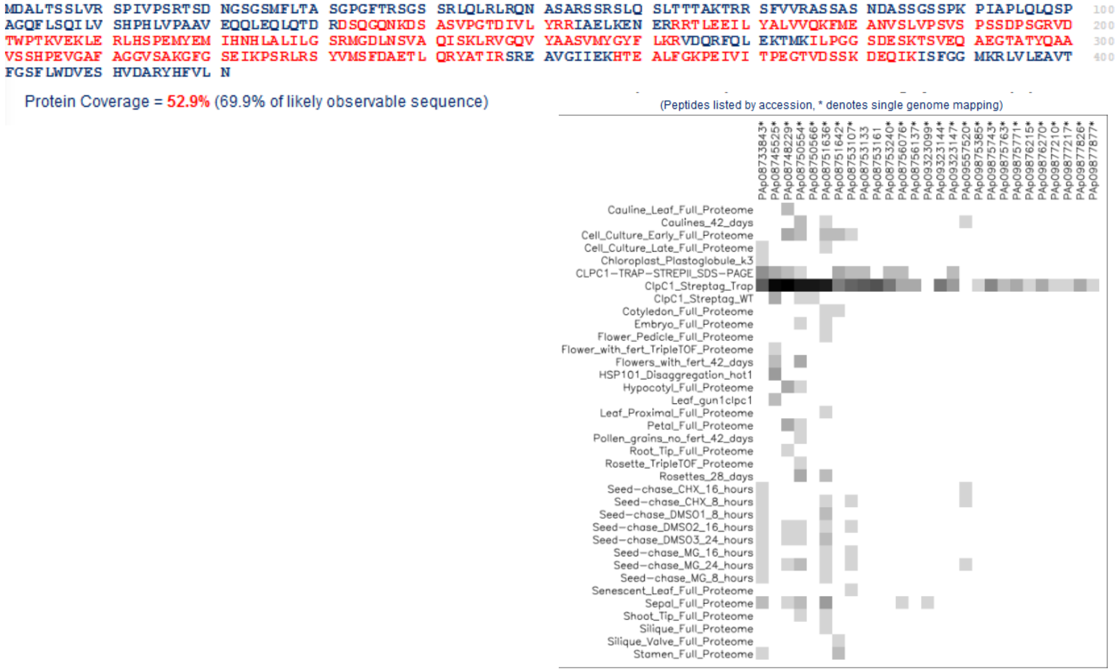

AT3G17800.1 – DUF760-6
start D131 after R
27 peptides, 467 observations.
36 experiments but by far the highest in CLPC1-Trap

Figure S6

MAAASARAFF MLSRVTDLSK KKLILHQPPP SSSPHRLPYA PNRAVSSSAV ISCLSGGGVS SDDSYVSTRR SKLDRGFAVI ANLVNRIQPL DTSVISKGLS 100
DSAKDSMKQT ISSMLGLLPS DQFSVSVTIS EQPLYRLLIS SIITGYTLWN AEYRVSLRRN FDIPIDPRKE EEDQSSKDNV RFGSEKGMSE DLGNCVEEFE 200
RLSPQVFGDL SPEALSYIQL LQSELSSMKE ELDSQKKKAL RIECEKGNRN DLLDYLRSLD PEMVTELSQL SSPEVEEIVN QLVQNVLERL FEDQTTSNFM 300
QNPGIRTTEG GDGTGRKVDT SRDYLAKLLF WCMLLGHHLR GLENRLHLSC VVGLL

Protein Coverage = 32.3% (36.2% of likely observable sequence)

Per-experiment expression for 40 most highly observed peptides

(Peptides listed by accession, * denotes single genome mapping)

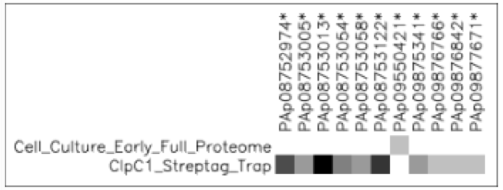

AT5G14970.1 - DUF760-7

start L54 after C53 -> **N-terminus**

11 peptides, 42 observations.

Only 2 experiments. **By far the highest in CLPC1-trap**, Cell culture

MDTSLSHHGS LPFPPSRRNF VKQNRGGDCV FLPSRRKFRY DSLVVVSAAS SGQSIDAPLV PRSPQGRFLS SVLVKKRQLF HFAVADLLKQ LADDKEASLS 100
RMFLSYGSDE ASLHRRIAQL KESDCQIAIE DIMYMLILYK FSEIRVPLVP KLPSCIYNGR LEISPSKDWE LESIHSFDVL ELIKEHSNAV ISLRVNSSLT 200
DDCATTEIDK NRLSKVYTAS VLYGYFLKSA SLRHQLECSL SQHHGSFTKQ LRHYISEFDP KILRRCAKPR SHEAKSLIEK QSLALFGPEE SSKESIVTSF 300
SSLKRLLLEA VAFGTFLWDT EEYVDGAFKL KENENAEEET NSSV

Protein Coverage = 0% (0% of likely observable sequence)

AT5G48590.1 -DUF760-8

Not detected

Figure S7

MALHGSGVFC KVSNMVEITS PFGGSMRLLH LPKSYPIHCN MVSASNTFGS AHLKLQNKEP CSRLRPCRVK REENNQTADV ESISMDENTL KQDLETAVQE 100
ENYVEAAKIR DKLKELQEDN KASVLSANSR FYQSFRNGDL AAMQSLWSKS GNPCCVHPGA KGITGYDYVM ESWELVWMNY EFPLLIELKD VEVHVRGEVG 200
YVTCMEFVKT KGSSSWGAQF VSNVFERIDG QWFICIHHAS PVDI

Protein Coverage = 55.7% (60.7% of likely observable sequence)

AT3G09250.1 - UVR1

start R71 after K.
13 peptides, 334 observations.
59 experiments; highest in leaf SEC, rosette & stems;
Very low in CLPC1-Trap – 1 peptide – not in other ClpC1 exp.
We did not identify with Mascot pipeline.

MRSVQAPVVC PAIRPRQVGA CASLVNYTGL KPRSQFWGNR TKGVKSQGTT TTITLRLCNK SIKCVFSSHS DGNGSTAENF NENDEEYVNS SVVEAVEVKS 100
GADGFMVKMR DGRQLRCVHN NPQGGHLPDY APHPAIVLKM EDGTGLLLPI IVLEMPSVLL MAAMTNVQIA RPTMYQVVKE MVDKMGYEVR LVRVTKRVHE 200
AYFAQLFLSK VGNASECVSF DLRPSDAINI AVRCKIPIQV NKYLAYSDGM RVIESGKIST PAPASDGLLF TEQDRPNGQA CLDTKEFNIL SKMMQAVDEE 300
RYDEAAEWRD KLGQFRAKRN LRKYT

Protein Coverage = 60% (68.9% of likely observable sequence)

AT1G75380.1 - UVR2

start V65 after C -> **N-terminus**
23 peptides, 403 observations, 50 experiments.
By far the highest in CLPC1-Trap

MRSLQAPVVC PSVRPRQLGV SALLVNCSVS KTRSLRKQFW GNQTKNDKSQ AATVNLRLHL RRYKSIKCLF SSHSDGTGST AENFNENDED YVKSSVLEAV 100
EVKSGPDGFM VKMKDGRQLR CVHNNPQGGN LPNYAPHSAI VLKMEDGTGL LLPIIVLEMP SVLLMAAMTN VQIARPTMYQ VVKDMVDKMG YEVRLVRVTT 200
RVHEAYFAEL YLSKVGDKSD CVSFDLRPSD AINIAVRCKV PIQVNKYLAY SDGMRVIDSG KLSKQTPASD GLLFTELDRP NGQPCFDTKE FDLVRNMMQA 300
VDEERYDEAA EWRDKLGKFQ AKRKLRKYT

Protein Coverage = 54.7% (66.4% of likely observable sequence)

AT1G19660.1 - UVR3

start L69 after C68 -> **N-terminus**
27 peptides, 307 observations,45 experiments
CLPC1-trap has most observations

MPGVAFSCVP ASDLGIFISE STTFSRSISA SLSSSSSSPS SFRCCTPLNL KSLRSHRRPK ISHICCDGSS ISGSGLGDPD LEFLQASVLV AETSMHYKMR 100
RHGFRQDSMW QTSRPLPPFS IRASESRVGV LPIGLGFLRQ FKQPTIFLKI SCDGDYLLPV IVGDAAVEKL LDVPLQGHTE ECPDQFQFVS AVVDKLGYEV 200
KMVKLTGRIV NTYYASLCLG KPGDIEAICI DSRPSDAINV ARACQAPIYV NKAIVLEEAI KIGYGGRPQS AKPVFNVILD SAPDGPDPLS EELKLVRNMD 300
LASKEERYID AAMWRDRLKN LQNSSSVVYN KGVETPESYE

Protein Coverage = 62.3% (65.8% of likely observable sequence)

(Peptides listed by accession, * denotes single genome mapping)

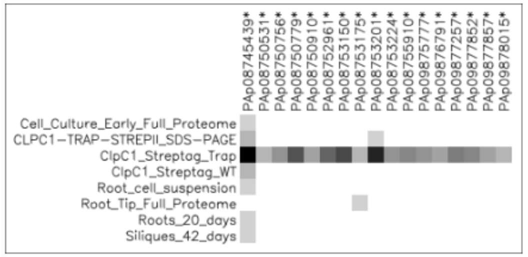

AT5G66050.1 – UVR4

start D67 (after C66) -> **N-terminus**
17 peptides, 156 observations.
Only 8 experiments. By far most in CLPC1-Trap
also Montandon et al (2019)

Figure S8

MAVPGFFSSM ALLRHCPVSN TEDGGGSFFH VAPRRTFRPH LLNTSSGKYL RRNRTQAIAE YLGSASDPKK PTGKSSYHPS EDIRAYVPEK NPGDSRLSPP 100
ETARTIIEVN KKGTLMLSGL LGIGVHENIL WPDIPYVTDQ HGNIYFQVKE NEDIMQTVVT SDNNYVQVIV GFDTMEMIKD MELSSPSGIG FGIEEIEDGE 200
SEVEDENKGD EDEGEDKDDE EWVAVLEDGD DEDNYVSDSD ESLGDWANLE TMRYCHPMYF ARRMAEVAST DPVNWMDQPS AGLAIQGLLS PVIVEDHSDI 300
QKHISGCIST GTDKNKEREN SEEIFEGIGE NESEILHVEN SRNAIQYYKL EIIRIQLITA QGHQTEVEVE DVRKAQPDVI ACASDGILTR LEEDGDKLTE 400
ALRSLCWRNN GIQAEEVKLI GIDSLGFDLR ICSGMQIETL RFAFSIRATS EHNAEGQLRE LLFASTPSKP QKPKQTNQKE S

Protein Coverage = 48.8% (51.8% of likely observable sequence)

AT5G24060.1 - HugZ1

start I58 after A57 -> **N-terminus**

24 peptides, 491 observations.

65 experiments but by far the highest in CLPC1-Trap

also CLPC1-Trap Montandon et al (2019). Not in CLPC-WT

MIESVMAVRL STGFCSSTAL LQYRTAPSSE EGGNCFHYAS RRVFQPQRIH HIDGSGFLKY NSDYITRKHL RKNRTQATAE YVDSASDPEK QTGKSRYHPS 100
EEIRASLPQN DGDSRLSPAE TTRTIIEVNN KGTLMLTGSI GDGVHENILW PDIPYITDQN GNLYFQVKED EDVMQSVTSE NNYVQVIVGF DTMEMIKEME 200
LMGLSDSDFE TEDDESGDDD SEDTGEDEDE EEWVAILEDE DEDDDDDDDD DEDDDDSDSD ESLGDWANLE TMRSCHPMFF AKRMTEVASN DPVDWMDQPS 300
AGLAIQGLLS HILVEDYSDI QKKLADSNST TNGNKDAENL VDKLEDNSKA GGDESEIDSS QDEKARNVVA FYKLEMIRIQ LITAQGDQTE VEVEDVRKAQ 400
PDAIAHASAE IISRLEESGD KITEALKSLC WRHNSIQAEE VKLIGIDSLG FDLRLCAGAK IESLRFAFST RATSEENAEG QIRKLLFPKT NQSTQPKPK

Protein Coverage = 42.8% (54% of likely observable sequence)

AT3G49140.1 - HugZ-2

start A78 after Q -> **N-terminus**

31 peptides, 901 observations,

pS355 phosphorylated – chloroplast p-proteome Schonberg et al 2017

108 experiments – many tissues (root, flower, embryo, etc)

Not particularly high in CLPC1-TRAP samples. Not in CLPC1-WT

MVIAAASSFS LGPSHCHQSY TDEFSSSIPY KRTSNARNRV FDGCGSANLS VLSSRCKIPF FGSAFHVSSG GHDLGLTKVS VAADYSDSVP DSSFYGYHPL 100
EDLKPSKRVQ ETKLSASEVA RTTVEANSSA VLVFPGAIHC EPHDHNSWSE FKYVIDDYGD IFFEIPDDEN ILEDPGASNP VKAFFGMDVP RYENTRHHEE 200
YNISDIGNLD QIIFDDHYFE IMDSEARDIP IDWGMPDTSN GVHPIYFAKH LSKAISMDYD RKMDYPSNGV SILGCLRPAF LDEESYIRRL FLSEDRDDYS 300
WEVQGDDNPI TSSRRDENDM SSSLYRLEIV GIELLSLYGA ESSISLQDFQ DAEPDILVHS TSAIIERFNN RGINSSIALK ALCKKKGLHA EEANLISVDS 400
LGMDVRVFAG AQVQTHRFPF KTRATTEMAA EKKIHQLLFP RSRRRKLKCH DESLKDAFR

Protein Coverage = 35% (39.4% of likely observable sequence)

(Peptides listed by accession, * denotes single genome

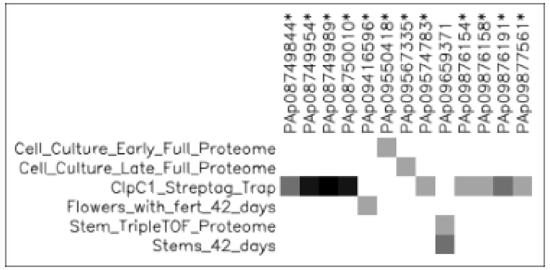

AT3G59300.1 - HugZ-3

start L49 after S48 -> **N-terminus**

13 peptides, 30 observation.

Only 6 experiments. 2x Cell culture, flower, stems, C1-trap

By far the highest in CLPC1-trap. Not in CLPC1-WT

Figure S9

```
MPSLSTPPSQ NLAFSPAASA TSSRLTPSSK RSFYPHRLPD PTALCRCSSS SGSNSSSSSS SDDNPRWDSA IQDVLKSAIK RFDSVLSWYA TLDNDDGEQG 100
SENVEKIDDD WDWDRWKKHF DQVDDQDRLL SVLKSQLNRA IKREDYEDAA RLKVAIAATA TNDAVGKVMS TFYRALLEER YKDAVYLRDK AGAGLVGWWS 200
GISEDVKDPF GLIVQITAEH GRYVARSYNP RQLSTSAAGA PLFEIFLTLD GKGNYKKQAV YLKWKEIFPD VPTMPSRTLT PGRFLTSPGR KEDTGNLAVE 300
SSEDEESDNS DDDSDLLEES SGFQSFLRDM IPGVKVKVMK VTAPGRVDKD FISKVIEQIA DEEDEENDLD IEDIDVEDDT KAEIDEKNAD IELESVTDEI 400
IDNNGGREIA VKFVIGDIVD RLSGNQPLKE SLRSPANLES VENSSFYLRL EKDLNVKESK GVEGTTLVDG KGSRQSRRRI ENIMGDLAKS IEKEKKISVK 500
MLKDVGELLS LTLSQAQNRQ QLSGLTKFRR IDVTPSLDPL DGLYIGAHGL YTSEVIHLKR KFGQWKGGKE SKKPTDIEFY EYVEAVKLTG DPYVPAGKVA 600
FRAKIGRRYE LPHKGLIPEE FGVIARYKGQ GRLADPGFRN PRWVDGELVI LDGKYVKGGP VVGFVYWAPE YHFVMFFNRL RLQA
```

Protein Coverage = 54.2% (64.2% of likely observable sequence)

AT4G33630.1 – EXE1

start W67 after R66

43 peptides, 844 observations, 84 experiments

by far the highest in CLPC1-Trap – not in CLPC1-WT

```
MATTQPCLIG QIIAVPQFHI LFSPRNSLKP ELSTNKRTNF SVSIGLRHSF ASSISTCNPK APSLSCLRNC AAVDGADTSS SEDKWDWDWD RWNRHFSEIE 100
EVESVVSLLK SQLEDAVEKE DFEEAVKLKQ AISEATVDDA VAEIMRQLQT AVNEERYHDA SRLCNETGSG LVGWWVGLPR DSEEPFGRIV HITPGVGRFI 200
GKSYSPRQLV AEAAGTPLFE IFVIKDTDGG YVMQVVYVQH VKQNLTISEN SFSKVQQSSK SSINDPSILD VRGSELKVDK KEDTQLNAGE PTEEGIKNVI 300
KFLKDKIPGL KLKVMDVIKI PEEEIVGSDD ATEELVGEGT EETNSSDDEE EVEEEENDSI EAISSMDSAD YGKHSNTKLV IGGVLHNIED SSIDDEIVRV 400
SANIMDTERD SFILHVPGRS KRDIDTRKNR VSKEQVTALA AQGLSDLLPP EVAEAFWGEK ASLKVSKHVH EIVKLAINQA QKGNHLSEYT AFNRIITPES 500
NLDPFDGLYV GAFGPYGTEI VQLKRKYGRW DDAEGSNSSD IEFFEYVEAV KLTGDPNVPA GQVTFRARIG NGSRMTNHGL FPEELGVLAS YRGQGKIADF 600
GFKKPRWVEG KLLKLNGKGM GPYVKGADLG FLYIGPEQSF LVLFNRLRLP E
```

Protein Coverage = 58.8% (73.6% of likely observable sequence)

AT1G27510.1 – EXE2

start H95 after R94

37 peptides, 926 observations, 74 experiments

CLPC1-trap has most observations

```
MSLSTIASGI GVLELPCLRL LPISSTQIQF LTVEARKQSR RIRRERSFSP FPVLIQSNRR LRHGFSELNS SFDRSNSGET GSDTTLKDGE EVRSESSSGV 100
GDSYVGLFVG MLGLDNDPLD REQAIETLWK YSLGGKKCID AIMQFHGCLN LIVNLLKSES SSACEAAAGL IRSIASVNLY RESVAESGAL EEITALLSRP 200
SLATVVKEQC ICALWNLTVD EEIREKVADF DILRLLISFL EDDDVNVKEA AGGVLANLAL SRSTHKILVE VGVIPKLAKL LKADNTENKG SKVIRKEARN 300
VLLELAKDEY YRILVIEEGV VPIPIIGADA YKSFRPDLYS WPSLPDGINI EQTAKAPSRF GASELLLGLN VDKNVDDVDE AKMKAIVGRT NQQFLARIGA 400
IEFEKEIKSE GPGKSQQLTL LPCVDGVARL VLILGLADEL AATRAAESIA DASINEDMRV SFMEAGAVKP LVQLLANNNK ETVKLPVIRA LKNLSLSRTV 500
CQRIEAEGAV WFLINLLKQP EISLNVTEHV LDIIAHILDP SKEMESKFYE GPVNGSKADS RKEVLDAAVF SRLVQIAKTA SPNLLRNAIS VIEFGIISNP 600
NMDTIISKDI TTVLDLALRQ KVLEEPENEA EELEKHLLKL EEAGLTISAA SRLLTKLLDS ESFRQTIDTA VFIELVRKIL RSSLPLHYKD WVAACLVKLT 700
ALSSPSQSLN NPINLEVTLY KTIPSLVEQM SFSSSPETKE AAVLELNKIV SEGVPESIQT LASQGGIEPL VKLLEERNER CVEASLSVLY NLTMDSENHT 800
AIIRAGAVPV LRRIVMSQRP QWEKALRLLR NLPV
```

Protein Coverage = 70% (77.9% of likely observable sequence)

AT1G23180.1 - ARM

S75 after R74 (single observation for M1)

61 peptides, 2833 observations, 108 experiments

Many peptides in exp. other than CLPC1-trap

```
MNCVSAFEFV PSSALFPCAQ SSIPAKFLHI LSSKASISPR CRSFASSSSS TIRRKPLTLV SSKSSDAEEV SDTEDEWLKK LPEKNKPLYS HSLPCIEAWL 100
RKLGFYQSKD DRAVWLIQKP DWHAQLSLDV TDLCIRYMKS GPGNLERDME RRFSYALSRE DTENAILGGP
```

Protein Coverage = 57.6% (62.8% of likely observable sequence)

AT5G52960.1 – DUF3143

start S62 (after S61) -> **N-terminus**

15 peptides, 833 observations, **pS65 & pT71 phosphorylation**

74 experiments – one very dominant p-peptide

R|SSDAEEVSDTEDEWLK

In both CLPC1-Trap and CLPC1-wt

p-peptide also in Schonberg 2017

Figure S10
